# Hierarchical Interconnected NiMoN with Large Specific Surface Area and High Mechanical Strength for Efficient and Stable Alkaline Water/Seawater Hydrogen Evolution

**DOI:** 10.1007/s40820-023-01129-y

**Published:** 2023-06-19

**Authors:** Minghui Ning, Yu Wang, Libo Wu, Lun Yang, Zhaoyang Chen, Shaowei Song, Yan Yao, Jiming Bao, Shuo Chen, Zhifeng Ren

**Affiliations:** 1 Department of Physics and Texas Center for Superconductivity at the University of Houston (TcSUH), University of Houston, Houston, TX 77204 USA; 2 Cullen College of Engineering and TcSUH, University of Houston, Houston, TX 77204 USA; 3 School of Materials Science and Engineering, Hubei Normal University, Huangshi, 435002 Hubei People’s Republic of China; 4 Department of Electrical and Computer Engineering and TcSUH, University of Houston, Houston, TX 77204 USA

**Keywords:** Hydrogen evolution reaction (HER), Nanoarchitecture, NiMo catalysts, Direct seawater electrolysis, Nanostructural stability

## Abstract

NiMo-based nanostructures are among the most active hydrogen evolution reaction (HER) catalysts under an alkaline environment due to their strong water dissociation ability. However, these nanostructures are vulnerable to the destructive effects of H_2_ production, especially at industry-standard current densities. Therefore, developing a strategy to improve their mechanical strength while maintaining or even further increasing the activity of these nanocatalysts is of great interest to both the research and industrial communities. Here, a hierarchical interconnected NiMoN (HW-NiMoN-2h) with a nanorod-nanowire morphology was synthesized based on a rational combination of hydrothermal and water bath processes. HW-NiMoN-2h is found to exhibit excellent HER activity due to the accomodation of abundant active sites on its hierarchical morphology, in which nanowires connect free-standing nanorods, concurrently strengthening its structural stability to withstand H_2_ production at 1 A cm^−2^. Seawater is an attractive feedstock for water electrolysis since H_2_ generation and water desalination can be addressed simultaneously in a single process. The HER performance of HW-NiMoN-2h in alkaline seawater suggests that the presence of Na^+^ ions interferes with the reation kinetics, thus lowering its activity slightly. However, benefiting from its hierarchical and interconnected characteristics, HW-NiMoN-2h is found to deliver outstanding HER activity of 1 A cm^−2^ at 130 mV overpotential and to exhibit excellent stability at 1 A cm^−2^ over 70 h in 1 M KOH seawater.

**Supplementary Information:**

The online version contains supplementary material available at 10.1007/s40820-023-01129-y.

## Highlights

- A hierarchical interconnected NiMoN (HW-NiMoN-2h) was successfully prepared based on a rational combination of hydrothermal and water bath processes.
- HW-NiMoN-2h exhibited high hydrogen evolution reaction (HER) activity due to its large specific surface area and good stability due to its enhanced mechanical strength.
- In 1 M KOH seawater, HW-NiMoN-2h delivered current density of 1 A cm^−2^ for HER at an overpotential of 130 mV and showed excellent stability over 70 h at 1 A cm^−2^.

**Supplementary Information:**

The online version contains supplementary material available at 10.1007/s40820-023-01129-y.

## Graphical abstract

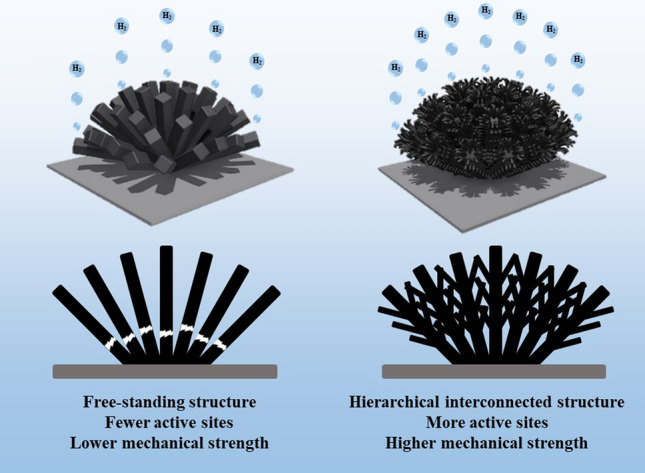

## Introduction

The hydrogen evolution reaction (HER) is one of the most studied electrochemical half-reactions since its product, H_2_, is widely regarded as a pivotal part of the future transition to clean energy [1]. Generally, HER is a three-phase reaction in which the H^+^ or H_2_O in the liquid electrolyte binds with the solid catalyst. Which then produces gaseous H_2_ [2, 3]. The phase transformation and the complex reaction mechanism inherent in the evolution from reactant to product introduce energy barriers to the reaction, so the development of a catalyst that can reduce such energy barriers and increase energy conversion efficiency during the reaction is of great interest to the academic and industrial communities [4–7].

The HER reaction process and mechanism vary under different environments and with different catalysts. In an acidic electrolyte, the first step of HER is the Volmer step, or the proton-adsorption step (* indicates the catalytic active site):

1 $$^{*} + {\text{H}}^{ + } + {\text{e}}^{ - } \to ^{*}{\text{H}}$$

In the Volmer-Tafel mechanism, the second step is the combination of two adjacent adsorbed protons:

2 $$2^{*} {\text{H }} \to 2^{*} + {\text{H}}_{2}$$

while in the Volmer-Heyrovsky mechanism, the second step is the adsorption of another proton on the adsorbed proton, followed by the desorption of H_2_:

3 $$^{*}{\text{H}} + {\text{H}}^{ + } + {\text{e}}^{ - } \to ^{*} + {\text{H}}_{{2}}$$

The simple reaction mechanisms in an acidic electrolyte are favorable to fast reaction kinetics [8, 9]. However, the corrosive nature of the acidic environment significantly limits active and stable catalysts to noble-metal-based materials, which increases costs and hinders the large-scale application of water electrolysis [10, 11]. In neutral and alkaline electrolytes, due to the lack of H^+^, the Volmer step becomes:

4 $$^{*} + {\text{H}}_{{2}} {\text{O}} + {\text{e}}^{ - } \to ^{*}{\text{H}} + {\text{OH}}^{ - }$$

The subsequent Tafel or Heyrovsky step is:

5 $${2}^{*}{\text{H}} \to { 2}^{*} + {\text{H}}_{{2}}$$

or

6 $$^{*}{\text{H}} + {\text{H}}_{{2}} {\text{O}} + {\text{e}}^{ - } \to ^{*} + {\text{H}}_{{2}} + {\text{OH}}^{ - }$$

respectively [12, 13]. Under neutral and alkaline environments, the mechanisms of HER involve two additional water dissociation processes as compared with those in an acidic environment, which is unfavorable to fast reaction kinetics [14, 15]. However, the relatively moderate characteristics of neutral and alkaline electrolytes allow the use of non-noble-metal catalysts and are thus beneficial to cost reduction in, and large-scale application of, water electrolysis [16, 17].

Among the various non-noble-metal HER catalysts, NiMo-based nanostructures are some of the most active in an alkaline environment [18–21]. Zhang et al*.* reported MoNi_4_ nanoparticles embedded on MoO_2_ cuboids on the surface of Ni foam and found that the nanocluster nature of MoO_2_ accommodates a tremendous amount of MoNi_4_, thus delivering HER activity of 10 mA cm^−2^ at merely 15 mV overpotential with a low Tafel slope of 30 mV dec^−1^ in 1 M KOH [19]. Further density functional theory (DFT) calculations showed that MoNi_4_ has a low energy barrier in the water dissociation process, which is normally the rate-determining step for HER in the alkaline condition. Wu et al*.* synthesized Ni-MoN nanowires on the surface of Ni foam, resulting in a high specific surface area of 27.5 m^2^ g^−1^ [14]. The Ni-MoN delivered a current density of 1 A cm^−2^ at a small overpotential of 136 mV and exhibited fast reaction kinetics with a small Tafel slope of 35.5 mV dec^−1^. The corresponding DFT simulation showed that Mo provides efficient water dissociation sites with a small H desorption energy barrier, thus contributing to the high HER activity of Ni-MoN. Therefore, the excellent water dissociation ability and high specific surface area of NiMo-based nanostructures make them among the best HER catalysts reported thus far.

Although nanostructured catalysts generally have higher specific surface areas and better intrinsic activity than their bulk counterparts, their mechanical strength remains a challenge, especially under an industrial-level current density [22–24]. Due to the generation of H_2_ bubbles during HER, a catalyst’s morphology will be deformed or even damaged under an industrial-level current density, which will significantly shorten its lifespan [25, 26]. A general solution is to enlarge the size of the nanostructure until an appropriate mechanical strength is obtained [27–29]. However, this is only a makeshift strategy since increasing the size of the nanostructure will result in the loss of a portion of the active sites or even decreased intrinsic activity [30, 31]. Therefore, manipulating the nanostructure to increase its mechanical strength while maintaining or even improving its catalytic activity for HER is of great importance for the industrial application of water electrolysis.

Here we synthesized a coral-like hierarchical interconnected Ni/Ni_0.2_Mo_0.8_N (NiMoN) on the surface of Ni foam (NF) using a hydrothermal process followed by a water bath process. The hierarchical structure maximizes the active sites on NiMoN and contributes to its state-of-the-art HER performance, in which the catalyst achieved a current density of 500 mA cm^−2^ at an overpotential of 76 mV and exhibited a small Tafel slope of 28.3 mV dec^−1^ in 1 M KOH. Results from sonication experiments and from chronopotentiometry tests at a large current density show that the interconnected structure strengthens the morphology of NiMoN. Therefore, the hierarchical interconnected NiMoN was found to outperform its free-standing counterparts prepared using only a hydrothermal or a water bath process in terms of both activity and stability. Since direct seawater electrolysis can address hydrogen production and seawater desalination simultaneously, thus attracting increasingly greater attention from the research and industrial communities, the HER performance of the hierarchical interconnected NiMoN was then systematically investigated in 1 M KOH seawater. Experiments showed that its excellent activity and stability are well preserved in 1 M KOH seawater, delivering a current density of 500 mA cm^−2^ at 91 mV overpotential and maintaining good stability at a current density of 1000 mA cm^−2^ over 70 h.

## Experimental Section

### Materials

Nickel (II) nitrate hexahydrate [Ni(NO_3_)_2_·6H_2_O, ≥ 97%, Sigma-Aldrich], ammonium molybdate tetrahydrate [(NH_4_)_6_Mo_7_O_24_·4H_2_O, 81.0%–83.0% MoO_3_ basis, Sigma-Aldrich], urea (Promega Corporation), sodium chloride (NaCl, Fisher Chemical), ethanol (C_2_H_5_OH, Decon Labs, Inc.), potassium hydroxide (KOH, 85%, pellets, ACS regent, Acros Organics), Pt/C (platium, nominally 20% on carbon black, Alfa Aesar), and hydrochloric acid (HCl, 36.5%–38.0% w/w, Fisher Chemical) were used without further purification. Ni foam (NF, thickness: 1.6 mm, porosity: ~ 95%) was used as the substrate for the preparation of all catalysts. NF was cleaned with 3 M HCl, ethanol, and deionized (DI) water several times before use. DI water was used to prepare solutions unless otherwise specified. Seawater was collected from Galveston Bay, Galveston, Texas, USA (29.303° N, 94.772° W) and was left standing for one week to allow the visible impurities to settle, after which the supernatant was collected before use. The white precipitates [mainly Ca(OH)_2_ and Mg(OH)_2_] produced during the preparation of alkaline natural seawater were removed by centrifugation at 7200 rpm for 5 min before use.

### Preparation of HT-NiMoO_4_ Nanorods

Free-standing HT-NiMoO_4_ nanorods were synthesized via a well-established hydrothermal (HT) method reported previously [32]. Briefly, a 50 mL solution with 0.04 M Ni(NO_3_)_2_·6H_2_O and 0.01 M (NH_4_)_6_Mo_7_O_24_·4H_2_O was prepared and then transferred into a 100 mL autoclave. A 2 cm × 5 cm piece of NF was transferred into the same autoclave. The autoclave was moved to a furnace for hydrothermal reaction at 150 °C for 6 h. After the reaction, the HT-NiMoO_4_ was sucessfully grown on the NF surface. The HT-NiMoO_4_/NF was cleaned with DI water several times and dried at 60 °C overnight under vacuum. Powdery HT-NiMoO_4_ was peeled from the NF surface via sonication for X-ray diffraction (XRD) analysis.

### Preparation of WB-NiMoO_4_ Nanowires

Free-standing WB-NiMoO_4_ nanowires were prepared based on a reported water bath (WB) method [14]. Briefly, a 20 mL solution with 0.1 M Ni(NO_3_)_2_·6H_2_O, 0.0125 M (NH_4_)_6_Mo_7_O_24_·4H_2_O and 0.15 M urea was prepared and then transferred into a 25 mL glass vial. A 1 cm × 3 cm piece of NF was transferred into the same vial. The vial was moved to a water bath oven and maintained at 90 °C for 8 h. After the water bath reaction, the WB-NiMoO_4_ was sucessfully grown on the NF surface, and the WB-NiMoO_4_/NF was rinsed with DI water several times and then dried at 60 °C overnight under vacuum. Powdery WB-NiMoO_4_ was peeled from the NF surface via sonication for XRD analysis.

### Preparation of HW-NiMoO_4_

The preparation of heirarchical interconnected HW-NiMoO_4_ was based on a rational combination of the aforementioned hydrothermal and water bath (HW) methods. First, the HT-NiMoO_4_/NF was prepared via the hydrothermal method described above. Subsequently, 1.5 cm × 2.5 cm pieces of HT-NiMoO_4_/NF were placed into vials with the solution as described above for the water bath method. For the water bath reaction, the temperature was set at 90 °C while the time varied from 1 to 4 h with 1 h intervals. The corresponding samples were denoted as HW-NiMoO_4_-1h, HW-NiMoO_4_-2h, HW-NiMoO_4_-3h, and HW-NiMoO_4_-4h. Powdery HW-NiMoO_4_-2h was peeled from the NF surface via sonication for XRD analysis.

### Nitridation of Different NiMoO_4_ Samples

The same nitridation process was employed to convert all the different NiMoO_4_ samples described above into NiMoN. Specifically, each sample was transferred into a tube furnace with a flowing gas of 120 standard cubic centimeters (sccm) NH_3_ and 30 sccm Ar. The thermal nitridation was conducted at 400 °C for 2 h. Afterward, the samples prepared from the original HT-NiMoO_4_/NF, WB-NiMoO_4_/NF, and HW-NiMoO_4_-*x*h (*x* = 1, 2, 3, and 4) samples were denoted as HT-NiMoN, WB-NiMoN, and HW-NiMoN-*x*h (*x* = 1, 2, 3, and 4), respectively.

### Preparation of Pt/C Electrode

40 mg Pt/C was dispersed in a mixture of 60 µL Nafion, 540 µL ethanol, and 400 µL DI water via sonication for 10 min. A small piece of NF was immersed into the mixture and sonicated for 1 h. Afterward, the NF with Pt/C coated on its surface was left on filter paper and dried at ambient conditions.

### Material Characterizations

Scanning electron microscopy (SEM) images were obtained using a LEO 1525 SEM. Transmission electron microscopy (TEM) images were obtained using a JEOL 2010F TEM. Inductively coupled plasma optical emission spectroscopy (ICP-OES) was obtained using an AGILENT 725 ICP-OES. X-ray diffraction (XRD) was conducted using a PANalytical X’pert PRO diffractometer with a Cu Ka radiation source. X-ray photoelectron spectroscopy (XPS) was performed using a PHI Quantera XPS scanning microprobe.

### Electrochemical Characterizations

Electrochemical measurements were performed on a Gamry Reference 600 electrochemical workstation. Experiments were conducted using a three-electrode configuration with a prepared sample (~ 0.5 cm^2^), a graphite electrode, and a Hg/HgO electrode serving as the working, counter, and reference electrodes, respectively. Cyclic voltammetry (CV) was measured at a scan rate of 2 mV s^−1^ with iR compensation [current-interrupt (CI) mode]. Potentials of the reference electrode were converted to the reversible hydrogen electrode (RHE) using the following equation:

7 $$E_{RHE} = E_{Hg/HgO} + 0.0{98} + 0.0{591} \times {\text{pH}}$$

The pH values of 1 M KOH, 1 M KOH 0.5 M NaCl, and 1 M KOH seawater electrolytes are all approximately 14.

### DFT Calculations

All spin-polarized DFT analyses were performed using the Vienna ab initio simulation package (VASP) code with the projector augmented wave (PAW) method [33, 34]. The generalized gradient approximation (GGA), combined with the Perdew-Burke-Ernzerhof (PBE) functional, was employed to describe the exchange–correlation term [35]. PAW pseudopotentials were used to describe the ionic cores [34]. A vacuum space of 15 Å along the *z*-axis was added to avoid interactions between the periodic slabs. The cutoff energy for the plane-wave basis was set to 450 eV. Van der Waals (VdW) interactions were described by using the empirical correction in Grimme’s scheme (DFT-D3) in all calculations [36]. The convergence tolerances for energy and force were set to 10^−5^ eV and 0.05 eV Å^−1^, respectively. The Gibbs free energy change (ΔG) for each elemental step was defined as:

8 $$\Delta {\text{G}} = \Delta {\text{E}}_{{{\text{DFT}}}} + \Delta {\text{E}}_{{{\text{ZPE}}}} - {\text{T}}\Delta {\text{S}}$$

where ΔE_DFT_ denotes the electronic energy change directly obtained from DFT calculations and ΔE_ZPE_ and ΔS are the zero-point energy correction and the entropy change, respectively, obtained from frequency calculations at 298.15 K.

## Results and Discussion

### Material Synthesis and Characterizations

**Fig. 1:**
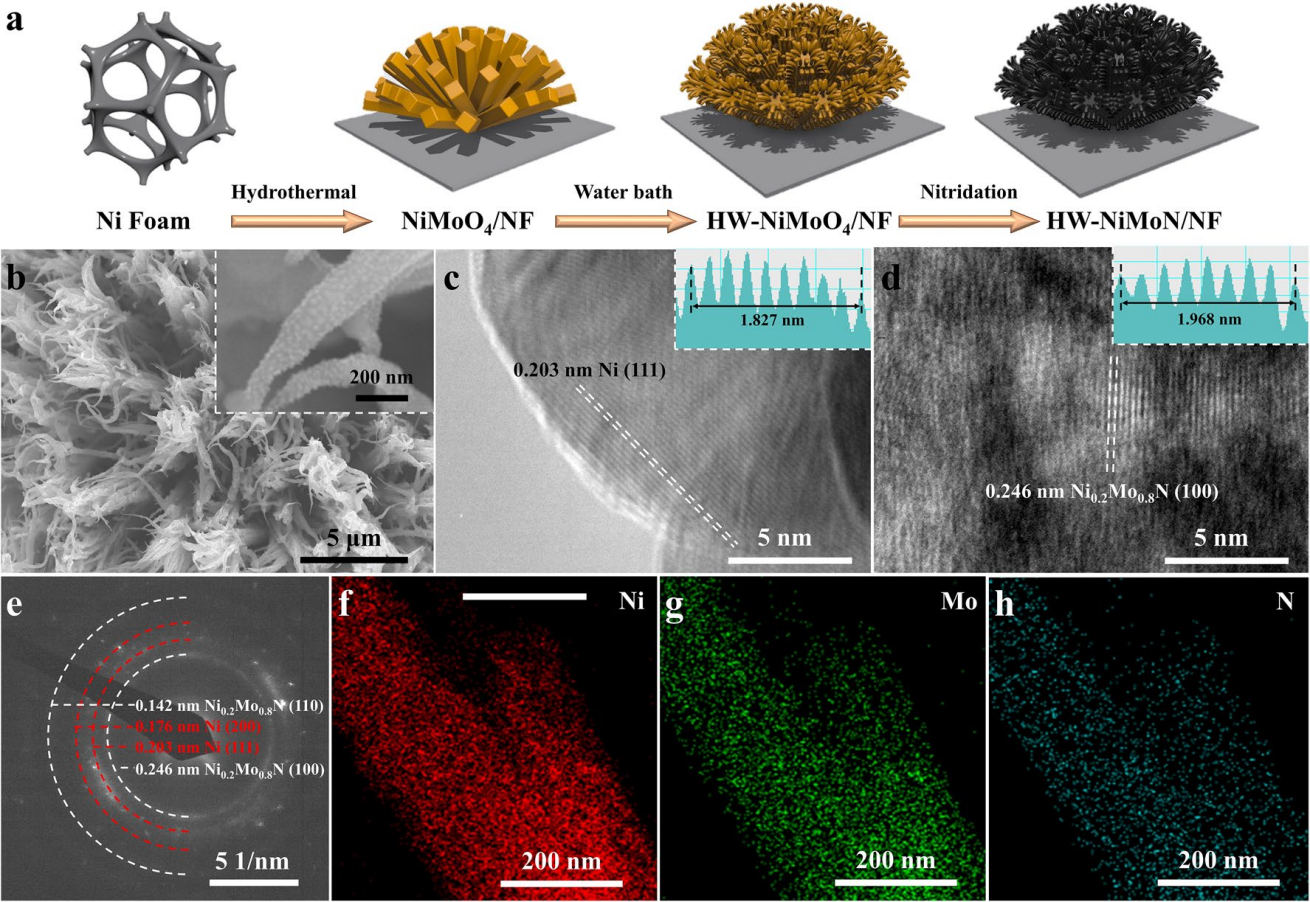
**a** Synthesis process for hierarchical interconnected HW-NiMoN on the surface of NF. **b** SEM images of HW-NiMoN-2h. **c, d** HRTEM images of HW-NiMoN-2h. **e** SAED of HW-NiMoN-2h. EDS mappings of **f** Ni, **g** Mo, and **h** N in HW-NiMoN-2h

The hierarchical interconnected HW-NiMoN was synthesized via the combination of hydrothermal and water bath reactions as illustrated in Fig. 1a. Briefly, NiMoO_4_ nanorods were grown on the surface of NF by a hydrothermal reaction and NiMoO_4_ nanowires of smaller size were then grown on the surfaces of NiMoO_4_ by a water bath reaction for different amounts of time to prepare the hierarchical interconnected HW-NiMoO_4_ [14, 32]. An image of HW-NiMoO_4_ prepared with different amounts of water bath reaction time is shown in Fig. S1. The color changes indicate that catalyst loading increases with increasing water bath time. The HW-NiMoO_4_ samples were subsequently converted into HW-NiMoN via thermal reduction and nitridation as described in the Experimental Section. HT-NiMoN and WB-NiMoN samples were additionally prepared as described in the Experimental Section and have free-standing nanorod and nanowire cluster structures, respectively, similar to that found in previous reports [14, 32]. Figures 1b and S2 show SEM images of all the HT-NiMoN, WB-NiMoN, and HW-NiMoN samples. A small amount of NiMoN nanowires can be observed on the surfaces of the NiMoN nanorods beginning with 1 h of water bath treatment. For HW-NiMoN-2h, NiMoN nanowires were uniformly grown on the surfaces of the NiMoN nanorods, creating a hierarchical interconnected structure as shown in Figs. 1b and S2g–i. The inset to Fig. 1b further illustrates the formation of nanodots on the surfaces of the NiMoN nanowires, and their presence is confirmed by the TEM image shown in Fig. S3. Images of HW-NiMoN-3 h and HW-NiMoN-4h in Fig. S2j–o show that further prolonging the water bath reaction time would introduce excessive NiMoN nanowires and completely fill the gaps within the NiMoN nanorod clusters, which might have the disadvantageous effect of reducing the active sites in the structure. Figure 1c, d shows high-resolution TEM (HRTEM) images of HW-NiMoN-2h. In Fig. 1c, the lattice space of the nanodot on the surface of the nanowire is 0.203 nm, which was calculated from the inset to Fig. 1c and corresponds to Ni (111). In Fig. 1d, the lattice space of 0.246 nm in the bulk of the nanowire, calculated from the corresponding inset, can be ascribed to Ni_0.2_Mo_0.8_N (100). The TEM images suggest that the nanodots on the surfaces of the NiMoN nanowires are mainly Ni nanoparticles. The selected area electron diffraction (SAED) pattern for HW-NiMoN-2h displayed in Fig. 1e shows the diffraction rings of Ni (200), Ni (111), Ni_0.2_Mo_0.8_N (110), and Ni_0.2_Mo_0.8_N (100). Energy dispersive X-ray spectroscopy (EDS) analysis was employed to study the elemental composition and distribution of HW-NiMoN-2h. The EDS point analysis shown in Fig. S4 indicates that the element ratio of Ni:Mo is approximately 1:1. The EDS mapping shown in Fig. 1f–h and the EDS linear scan shown in Fig. S5 suggest the uniform distribution of Ni, Mo, and N throughout the nanowires and nanorods of HW-NiMoN-2h. ICP-OES was further conducted to determine the amounts of individual metals in HW-NiMoN-2h. As shown in Table S1, the weight percentages of Ni and Mo were found to be 28.35% and 41.65%, respectively, so the atomic ratio of Ni–Mo is about 1.1:1.

**Fig. 2:**
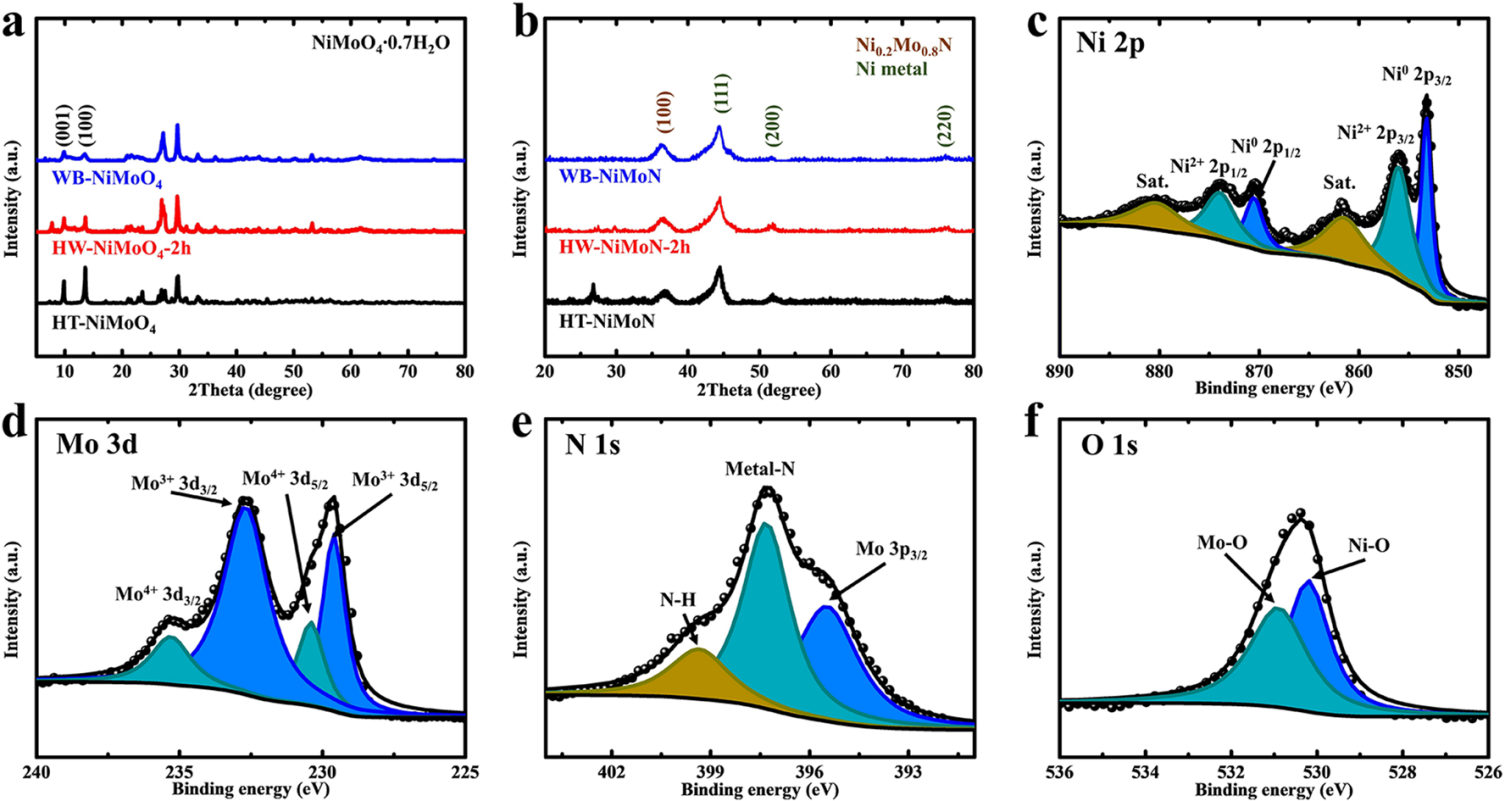
**a** XRD patterns for HT-NiMoO_4_, HW-NiMoO_4_-2h, and WB-NiMoO_4_. **b** XRD patterns for HT-NiMoN, HW-NiMoN-2h, and WB-NiMoN. XPS spectra of **c** Ni 2*p*, **d** Mo 3*d*, **e** N 1*s*, and **f** O 1*s* for HW-NiMoN-2h

XRD was further employed to study the crystal structure of the powdery samples. As shown in Fig. 2a, both HT-NiMoO_4_ and WB-NiMoO_4_ display the typical XRD pattern of NiMoO_4_∙0.7H_2_O (PDF#97-024-7435) [37, 38]. Due to the large diameter of HT-NiMoO_4_, its XRD pattern shows stronger intensities than that for WB-NiMoO_4_ at 9.83° and 13.577°, corresponding to the (001) and (100) planes, respectively, of NiMoO_4_∙0.7H_2_O. The similarity in crystal structure between HT-NiMoO_4_ and WB-NiMoO_4_ provides abundant sites to facilitate the growth of NiMoO_4_ nanowires on the surfaces of the NiMoO_4_ nanorods, and therefore explains the quick formation of the hierarchical interconnected structure of HW-NiMoO_4_-2h in comparison to the 8-h random growth of WB-NiMoO_4_ nanowire clusters on the surface of NF. The XRD pattern for HW-NiMoO_4_-2h is similar to that for WB-NiMoO_4_ but with slightly stronger intensities at 9.83° and 13.577°, which were inherited from HT-NiMoO_4_. Figure 2b shows the XRD patterns for HT-NiMoN, HW-NiMoN-2h, and WB-NiMoN, indicating that, regardless of the preparation method employed for any NiMoN sample, its XRD pattern exhibits four peaks of Ni_0.2_Mo_0.8_N (100), Ni (111), Ni (200), and Ni (220) (PDF#29-0931, PDF#04-0850) [39]. The XRD patterns for the NiMoN samples are consistent with the SAED pattern shown in Fig. 1e and verify that they are mainly composed of Ni and Ni_0.2_Mo_0.8_N. The additional peak at 27.339° in the XRD pattern for HT-NiMoN is contributed by the (021) plane of MoO_3_ (PDF#35-0609), indicating the incomplete nitridation of HT-NiMoN.

XPS spectra were obtained to investigate the valence states of elements in HW-NiMoN-2h. The survey spectrum shown in Fig. S6 exhibits peaks corresponding to Ni 2*p*, Mo 3*d*, N 1*s* and O 1*s*, which are shown in greater detail in the high-resolution spectra in Fig. 2c–f, respectively. In Fig. 2c, the peaks located at 853.2 and 870.5 eV can be ascribed to Ni^0^ 2*p*_3/2_ and Ni^0^ 2*p*_1/2_, respectively [32]. The peaks at 856.0 and 873.9 eV corresponding to Ni^2+^ 2*p*_3/2_ and Ni^2+^ 2*p*_1/2_, respectively, are caused by mild surface oxidation, and two satellite peaks can be observed at 861.5 and 880.3 eV [32]. The Mo 3*d* spectrum in Fig. 2d shows peaks at 229.6 and 232.7 eV corresponding to Mo^3+^ 3*d*_5/2_ and Mo^3+^ 3*d*_3/2_, respectively, which are contributed by the Mo–N bonds [14]. The peaks at 230.4 and 235.3 eV corresponding to Mo^4+^ 3*d*_5/2_ and Mo^4+^ 3*d*_3/2_ originate from the mild oxidation of HW-NiMoN-2h [32, 40]. The N 1*s* spectrum shown in Fig. 2e exhibits a metal-N peak at 397.3 eV and a N–H peak at 399.3 eV contributed by the adsorbed NH_3_, while the peak at 395.5 eV corresponds to Mo 2*p*_3/2_ of elemental Mo [18]. The presence of O 1*s* results from slight surface oxidation, and its spectrum shown in Fig. 2f exhibits Ni–O and Mo–O peaks at 530.2 and 530.9 eV, respectively [32]. The XPS measurements thus suggest that HW-NiMoN-2h is mainly composed of Ni metal and Ni_0.2_Mo_0.8_N and that mild oxidation occurred due to short exposure to the air.

### HER Performance in Alkaline Fresh Water

**Fig. 3:**
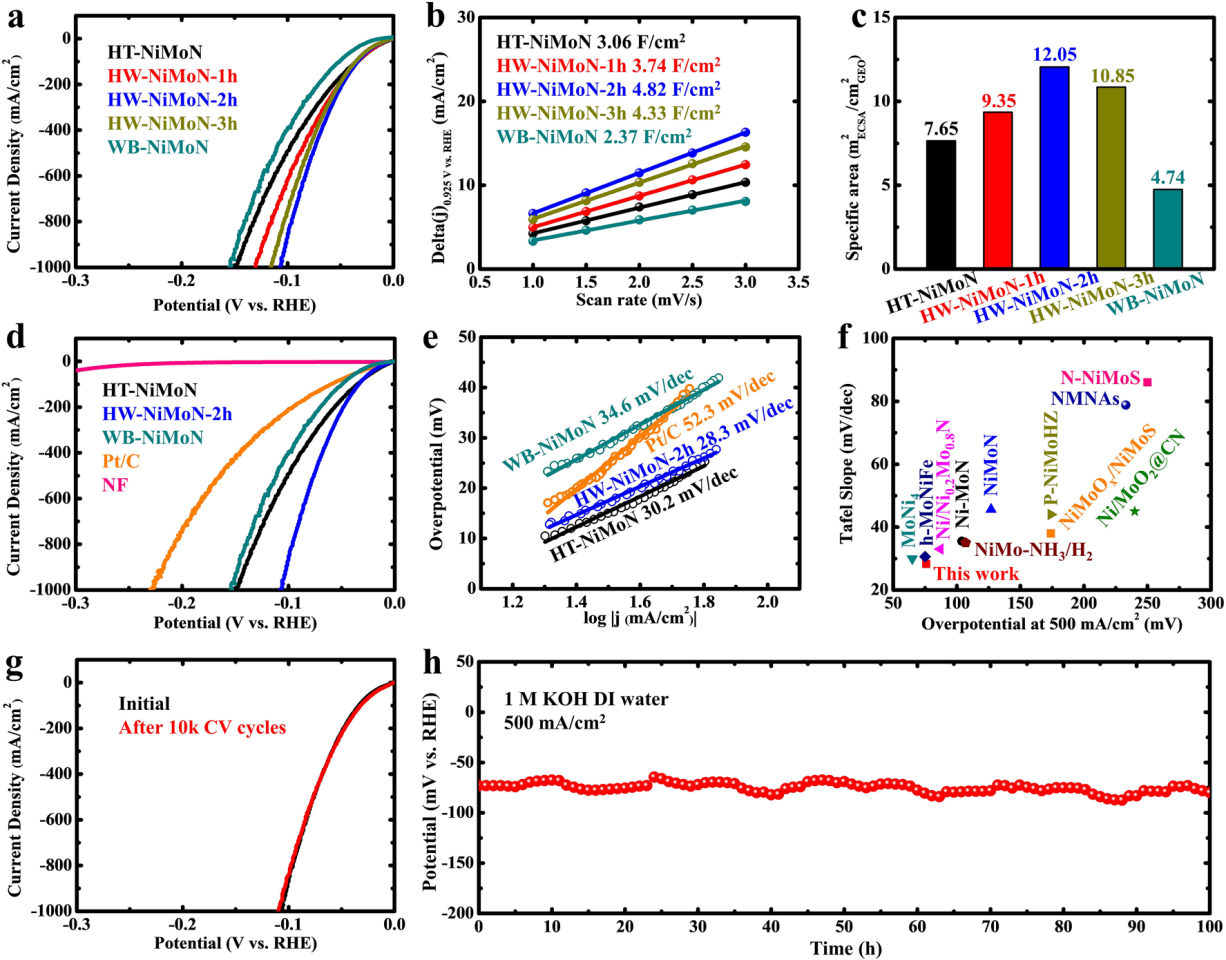
**a** Comparison of HER performance in 1 M KOH DI water among NiMoN samples prepared using different methods. **b** *C*_dl_ values of NiMoN samples prepared using different methods. **c** ECSA values of NiMoN samples prepared using different methods. **d** Comparison of HER performance in 1 M KOH DI water among different catalysts. **e** Tafel slopes for different catalysts. **f** Comparison of HER activity in 1 M KOH DI water between HW-NiMoN-2h and other state-of-the-art NiMo-based catalysts. **g** HER performance of HW-NiMoN-2h before and after 10,000 CV cycles. **h** Chronopotentiometric testing of HW-NiMoN-2h at 500 mA cm^−2^ in 1 M KOH DI water. The fluctuation over time results from changes in the water level and the ambient temperature

A comparison of HER performance in 1 M KOH DI water among NiMoN samples prepared using different methods is shown in Fig. 3a. All the HW-NiMoN catalysts exhibited better performance than either HT-NiMoN or WB-NiMoN. The HER performance of the HW-NiMoN samples increased with increasing water bath time and reached the optimal duration at 2 h, as shown in Fig. 3a. Electrochemical surface area (ECSA) analysis was employed to characterize the active area of selected NiMoN samples electrochemically [41]. Based on the CV measurements in Fig. S7, the ∆(j) of each sample was plotted as a function of the scan rate and their double layer capacitance (*C*_dl_) values were calculated, as shown and listed, respectively, in Fig. 3b. Based on the *C*_dl_ values, the corresponding ECSA values were calculated via the following equation:

9 $${\text{ECSA}} = C_{{{\text{dl}}}} /C_{{\text{s}}}$$

where *C*_s_ = 40 μF cm^−2^ [42], and the results are shown in Fig. 3c. The ECSA of the HW-NiMoN samples increased with increasing water bath time and reached the optimal duration at 2 h, which proves that HW-NiMoN-2h has the highest electrochemically active surface area among these catalysts. The turnover frequency (TOF) values of selected NiMoN samples were subsequently calculated based on the previously reported method to unveil their intrinsic HER activity [14, 43]. As respectively shown in Fig. S8a-b, the NiMoN samples exhibit similar TOF plots and values, and are thus composed of similar active species. The Brunauer–Emmett–Teller (BET) method was further utilized to study the specific surface area of NiMoNs from the physical adsorption perspective. The BET measurements and the corresponding BET area and mass density values for selected NiMoN samples are presented in Fig. S9 and Table S2. BET specific surface area is calculated via the multiplication of corresponding BET area and mass density values to represent the ratio of the catalyst’s BET area to its geometric area and, as displayed in Fig. S9f, the BET specific surface area values of the selected NiMoN samples show a trend similar to that for their HER performance and ECSA, with HW-NiMoN-2h exhibiting the highest value in each. Normalization of the NiMoNs’ HER performance by BET specific area was further calculated and the results are shown in Fig. S10. The WB-NiMoN and HT-NiMoN samples exhibit higher normalized activity than the HW-NiMoN samples, which indicates better utilization of actives sites on the catalysts with smaller BET specific area. However, based on Figs. S8 and 3a, the intrinsic activity of each individual active site is similar among different NiMoNs, and HW-NiMoN-2h has the highest total amount of accessible active sites. Contact angle tests were further conducted to investigate bubble release among the catalysts studied. As shown in Fig. S11, pure NF exhibited a large contact angle of 117° due to its hydrophobic nature, while no contact angle could be observed on any of the NiMoNs, clearly showing the highly hydrophilic characteristics of NiMoN. Therefore, the above analyses prove that the hierarchical HW-NiMoN structure can effectively increase the active surface area and then further improve the HER performance of the intrinsically highly active NiMoN catalyst.

The activity of selected NiMoN samples in 1 M KOH DI water was then compared with that of the benchmark Pt/C and bare NF, and the results are shown in Fig. 3d. HW-NiMoN-2h delivered current densities of 100, 500, and 1000 mA cm^−2^ at overpotentials of 34, 76, and 107 mV, respectively, while Pt/C required overpotentials of 60, 165, and 230 mV to drive the same respective current densities. The corresponding overpotentials required by HT-NiMoN, WB-NiMoN, and NF are summarized in Table S3. Tafel slopes determined from the results shown in Fig. 3d were utilized to investigate the HER kinetics of the various catalysts. As shown in Fig. 3e, the Tafel slopes for HT-NiMoN, HW-NiMoN-2h, and WB-NiMoN are 30.2, 28.3, and 34.6 mV dec^−1^, respectively. The Tafel slopes for the selected NiMoN samples are all close to 30 mV dec^−1^, indicating that the NiMoN synthesis follows the Volmer-Tafel mechanism and that the Tafel step, the combination of two adsorbed protons, is the rate-detemining step [44, 45]. In comparison, Pt/C and NF exhibited slower HER kinetics, with larger Tafel slopes of 52.3 and 139 mV dec^−1^, as shown in Figs. 3e and S12, respectively. The overpotential at 500 mA cm^-2^ of the HW-NiMoN-2h sample and its Tafel slope were then compared with those of other state-of-the-art NiMo-based HER catalysts. As shown in Fig. 3f, HW-NiMoN-2h is among the best HER catalysts, even in comparison with the highly active NiMo-based catalysts reported thus far [14, 18–21, 46–51].

In addition to its activity, the stability of HW-NiMoN-2h was also systematically investigated since a catalyst’s durability during H_2_ production is another critical criterion for determining its competence. Here stability was evaluated via repeated CV scans (from 0 to 0.15 V vs. RHE) and chronopotentiometric (CP) testing. As shown in Fig. 3g, the linear sweep voltammetry (LSV) curve for HW-NiMoN-2h after 10,000 CV scans shows no obvious decay compared to that before aging. The CP test results displayed in Fig. 3h show that the HW-NiMoN-2h sample experienced no obvious degradation during operation at 500 mA cm^−1^ for 100 h. To further confirm the stability of HW-NiMoN-2h, SEM and XPS analyses were conducted following the 100 h CP test. The SEM images displayed in Fig. S13a, b show that the morphology of HW-NiMoN-2h was well maintained even after 100 h of CP testing at 500 mA cm^−1^. The XPS spectra shown in Fig. S13c-f reveal that the surface of HW-NiMoN-2h was oxidized after testing for 100 h; specifically, the elemental Ni and Mo were oxidized to the + 2 and + 6 valence states, respectively, and the N 1*s* peaks disappeared [32]. Even though the XPS data indicated that the HW-NiMoN-2h surface was oxidized, the catalyst’s activity was well preserved, as shown by the repeated CV scans and CP testing.

**Fig. 4:**
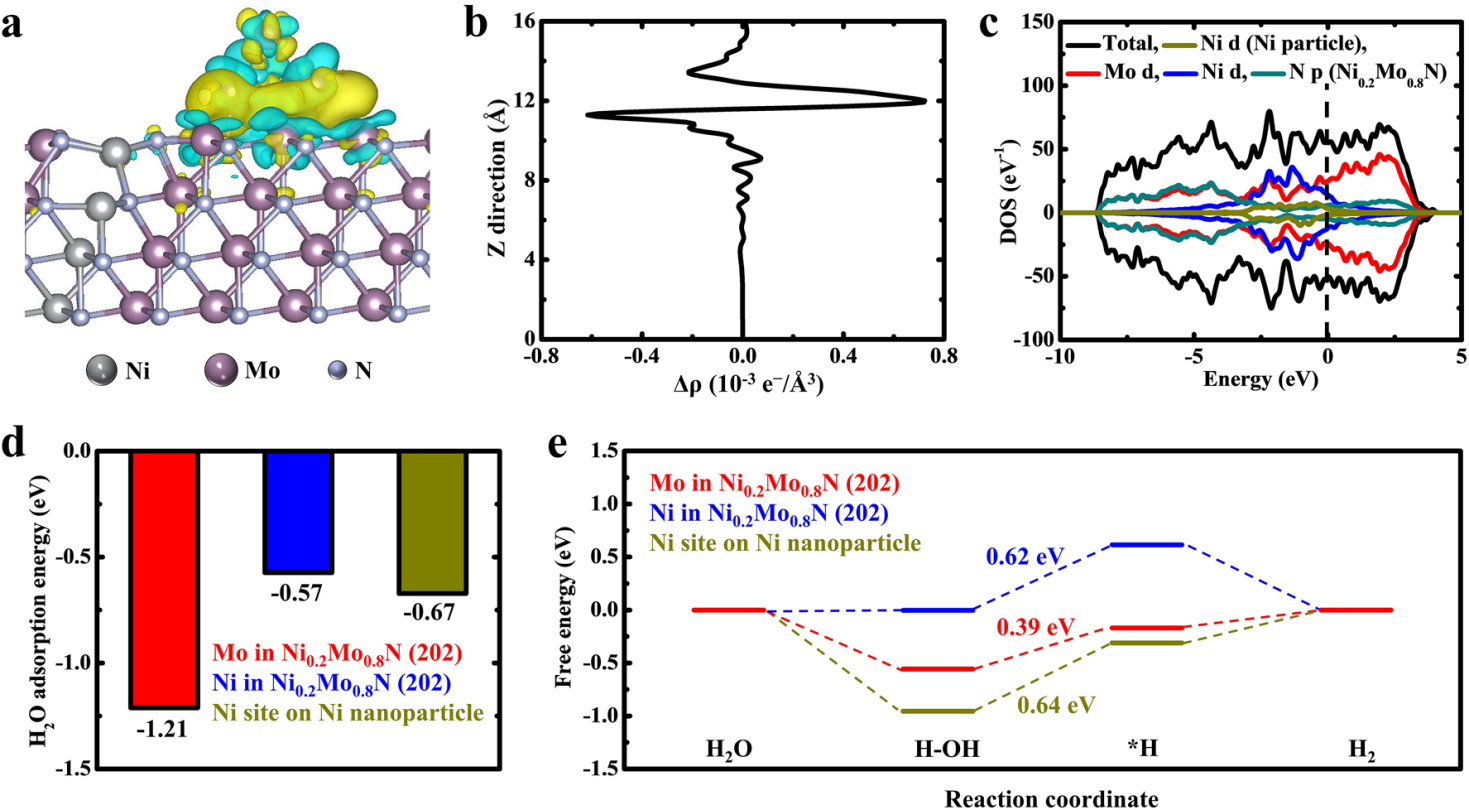
**a** Charge density differences in HW-NiMoN-2h. Yellow and cyan regions represent electron accumulation and depletion, respectively. **b** 1D planar-averaged charge density. **c** DOS of Mo, Ni, and N in Ni_0.2_Mo_0.8_N, and Ni in Ni nanoparticles. **d** H_2_O adsorption energy among different metal active sites on HW-NiMoN-2h. **e** Adsorption Gibbs free energy diagram for the HER pathway on different active sites of HW-NiMoN-2h

DFT calculations were performed to further investigate the HER active sites in HW-NiMoN-2h. The charge density differences shown in Fig. 4a reveal a strong electron accumulation at the heterointerface between the Ni metal cluster and Ni_0.2_Mo_0.8_N (202). The one-dimensional (1D) planar-averaged charge density shown in Fig. 4b further confirms the strong charge exchange between Ni and Ni_0.2_Mo_0.8_N. In Fig. 4c, the distribution of the density of states (DOS) suggests the high electronic conductivity of HW-NiMoN-2h, which is one of the prerequisites for a highly active HER catalyst. In an alkaline environment, the water dissociation process (*H_2_O + e^−^  → *H + OH^−^) and the subsequent *H adsorption/desorption energy are critical indicators for the activity of active sites. The H_2_O adsorption energy values among the different metal active sites on HW-NiMoN-2h were calculated and the results are shown in Fig. 4d. The Mo sites on Ni_0.2_Mo_0.8_N (202) were found to exhibit the lowest H_2_O adsorption energy, revealing its strong H_2_O adsorption ability, which is favorable for the later H_2_O dissociation process. The free energy diagram in Fig. 4e shows that the water dissociation step is the potential-determining step (PDS) and that the Mo sites display the smallest energy barrier of 0.39 eV at the PDS among the metal active sites on HW-NiMoN-2h. Therefore, the DFT calculations suggest that the Mo sites on Ni_0.2_Mo_0.8_N (202) are the active sites in HW-NiMoN-2h for HER due to their strong water adsorption capability and small energy barrier for water dissociation.

**Fig. 5:**
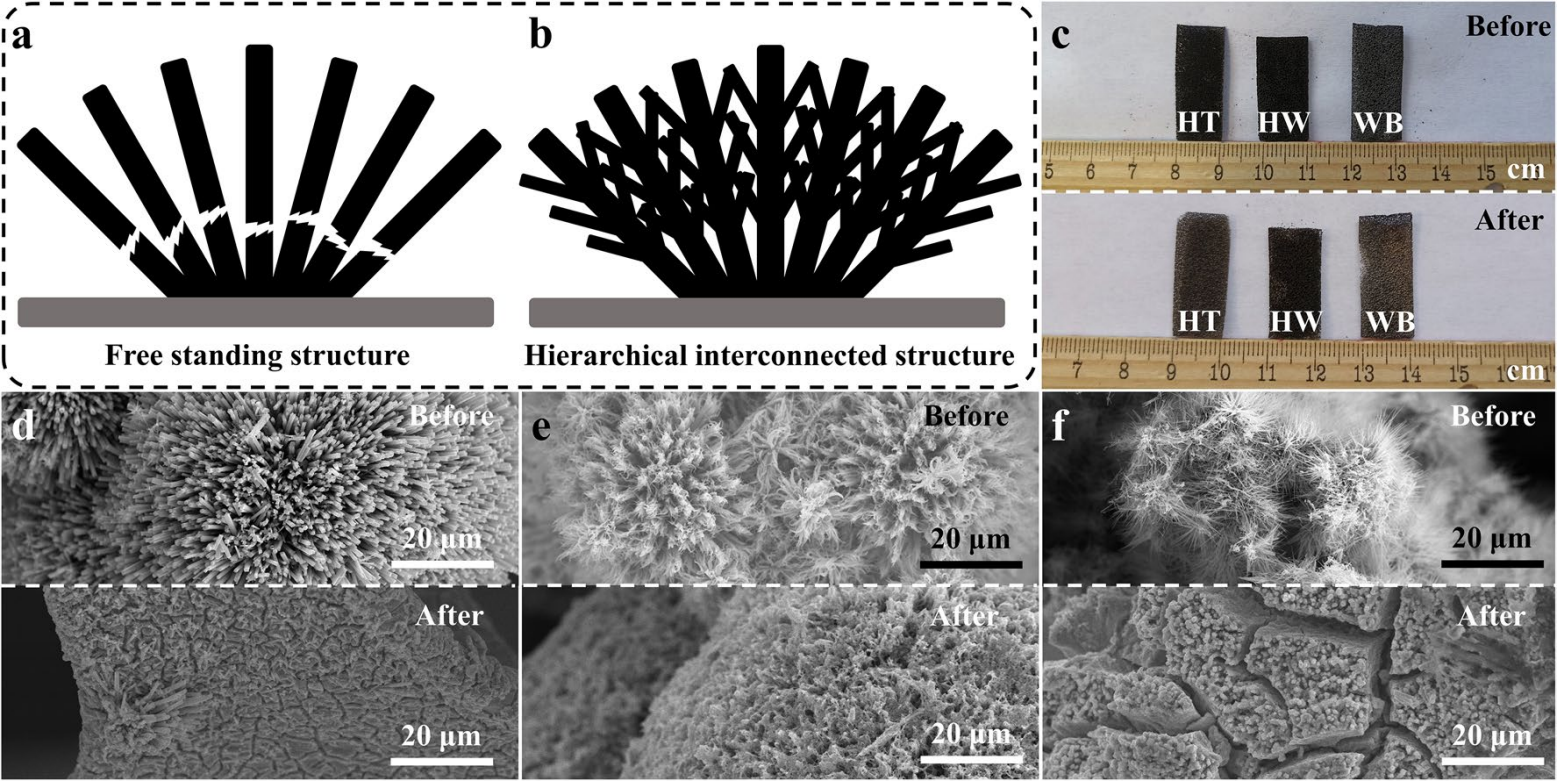
Illustrations of **a** free-standing and **b** hierarchical interconnected nanocatalyst structures. **c** Images of HT-NiMoN, HW-NiMoN-2h, and WB-NiMoN before and after 30 min sonication. SEM images of **d** HT-NiMoN, **e** HW-NiMoN-2h, and **f** WB-NiMoN before and after 30 min sonication

The mechanical strength of a catalyst is a critical component of its long-term stability [52–54]. Under an industrial-level current density such as 1 A cm^−2^, the rapid production of H_2_ bubbles places a severe demand on a nanocatalyst. In particular, it would be difficult for a free-standing nanowire or nanorod cluster, as shown in Fig. 5a, to withstand the tension caused by the quick generation and release of bubbles. A common solution is to enlarge the nanocatalyst size, sacrificing a portion of the specific surface area to strike a balance between activity and stability. Here we propose a hierarchical interconnected structure to unify the different free-standing structure components as shown in Fig. 5b, which not only increases the nanocatalyst’s active area but also improves its mechanical strength simultaneously. Here we evaluated the mechanical strength of selected prepared catalysts, first by an accelerated mechanical strength test, in which each sample was immersed in water and sonicated for 30 min. As shown in Fig. 5c, the metallic silver color of NF began to appear on the free-standing HT-NiMoN and WB-NiMoN samples after 30 min sonication, while the HW-NiMoN-2h sample maintained its deep dark NiMoN color. Similar phenomena can also be observed for the NiMoO_4_ precursors, as shown in Fig. S14. To confirm the structural integrity of the samples, SEM images were obtained following sonication. As shown in Figs. 5d and S15a–c, most of the HT-NiMoN nanorod clusters were peeled off after sonication. As shown in Figs. 5e and S15d–f, even though the HW-NiMoN-2h tips were broken, most of its hierarchical interconnected structure was preserved after sonication. Additionally, the SEM images displayed in Figs. 5f and S15g–i show that the nanowire structure of WB-NiMoN was destroyed by the sonication. Therefore, the accelerated mechanical strength tests revealed that the hierarchical interconnected structure has better mechanical strength than the free-standing structure. SEM images of different NiMoO_4_ samples after sonication are provided in Fig. S16 to confirm the high mechanical strength of the interconnected NiMoO_4_. To further confirm the above conclusion, selected NiMoN samples were tested under 1 A cm^−2^ in 1 M KOH DI water to study the mechanical strength of each under an industrial-level current density. As shown in Fig. S17, HT-NiMoN experienced quick degradation from − 0.149 to − 0.215 V versus RHE at 1 A cm^−2^ over 24 h of testing. The SEM images shown in Fig. S18a-c indicate that only a small portion of the NiMoN nanorod clusters were preserved on the HT-NiMoN sample following 24 h of CP testing. Additionally, the overpotential required by WB-NiMoN increased by 186 mV during 23 h of testing at 1 A cm^−2^ (Fig. S17) and its nanowire structure was almost completely destroyed as shown by the SEM images displayed in Fig. S18g–i. In contrast, the performance of HW-NiMoN-2h remained stable under the same condition, exhibiting a negligibly increased overpotential of 8 mV after 24h (Fig. S17). The morphology of HW-NiMoN-2h was well maintained after the CP test as shown in Fig. S18d-f. Excellent stability is also observed for HW-NiMoN-3h and HW-NiMoN-4h in Fig. S17, which again verifies the high mechanical strength of the interconnected structure and its significant role in the long-term stability of the nanocatalyst at industrial-level current density.

### HER Performance in Alkaline Seawater

**Fig. 6:**
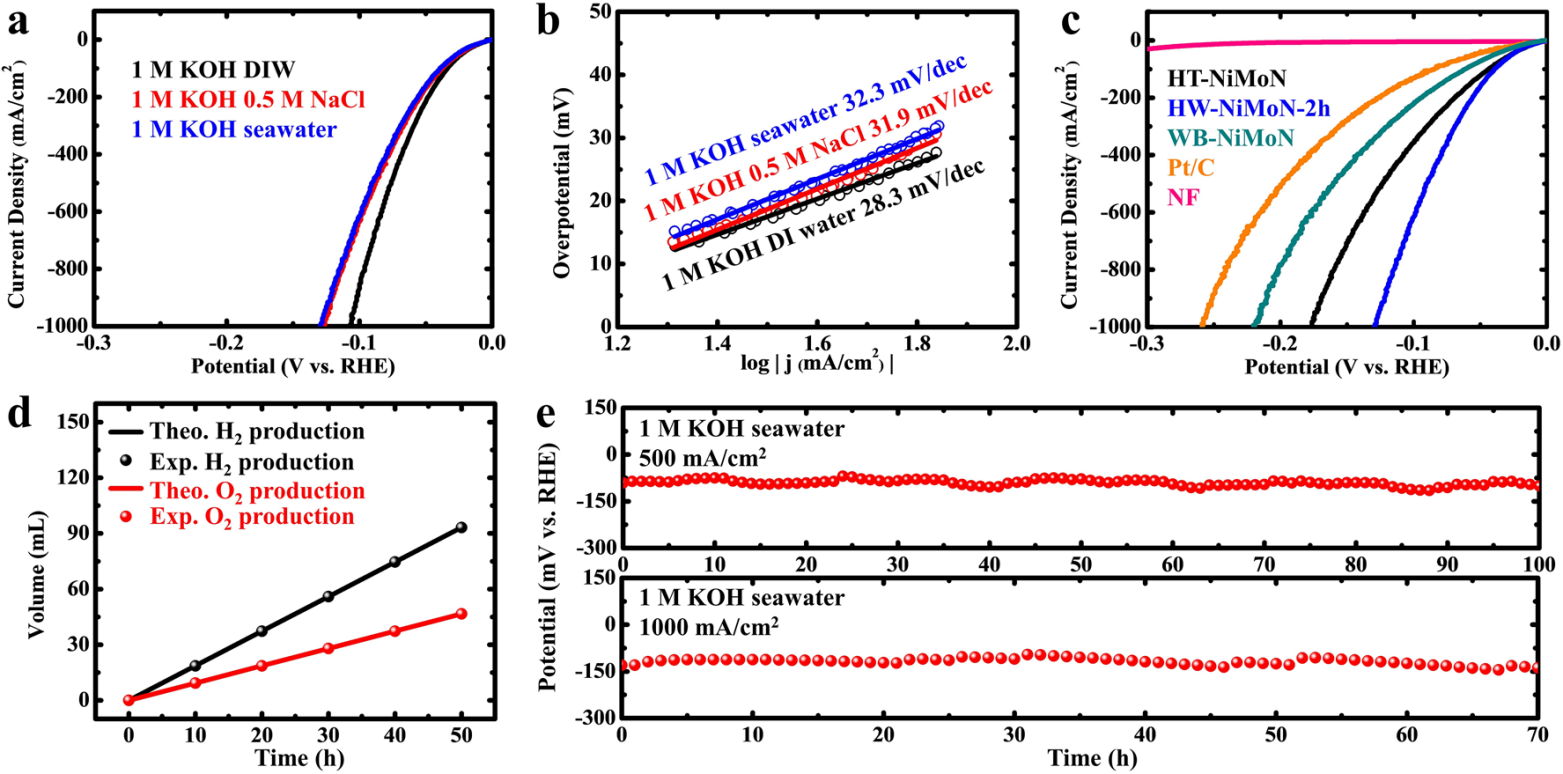
**a** HER performance of HW-NiMoN-2h in different electrolytes. **b** Tafel slopes for HW-NiMoN-2h in different electrolytes. **c** HER performance of different catalysts in 1 M KOH seawater. **d** FE of HW-NiMoN-2h||NiFe LDH at 500 mA cm^−2^ in 1 M KOH seawater. **e** Chronopotentiometric tests of HW-NiMoN-2h at 500 and 1000 mA cm^−2^ in 1 M KOH seawater

For large-scale H_2_ production on the order of hundreds of megawatts, the huge water consumption required will be a problem since fresh water remains a scarce resource in many areas around the world [55, 56]. Direct seawater H_2_ production is a promising strategy to overcome the conflict between fresh water shortages and the requirements for large-scale water electrolysis since seawater comprises about 97% of the water resources on earth [57, 58]. Here we systematically analyzed the HER performance of the prepared catalysts under the seawater condition was to gain further insight into seawater HER. In an alkaline environment, Ca^2+^ and Mg^2+^ ions in the seawater will precipitate as Ca(OH)_2_ and Mg(OH)_2_, as shown in Fig. S19. In this study, all Ca(OH)_2_ and Mg(OH)_2_ precipitates in the 1 M KOH seawater elecytrolyte were removed prior to electrochemical testing. Figure 6a shows the HER performance of HW-NiMoN-2h in 1 M KOH DI water, 1 M KOH 0.5 M NaCl, and 1 M KOH seawater. Its activity in 1 M KOH 0.5 M NaCl exhibited a small decrease compared to that in 1 M KOH DI water, suggesting that the addition of Na^+^ had a slightly negative effect on the HER performance [59, 60]. The HW-NiMoN-2h sample required overpotentials of 40, 91, and 130 mV to deliver current densities of 100, 500, and 1000 mA cm^−2^, respectively, in 1 M KOH seawater, close to its performance in 1 M KOH 0.5 M NaCl, as shown in Table S4. The Tafel slopes of HW-NiMoN-2h were then determined to study its reaction kinetics in different electrolytes. As shown in Fig. 6b, HW-NiMoN-2h exhibits very similar Tafel slopes in 1 M KOH 0.5 M NaCl and 1 M KOH seawater, with values of 31.9 and 32.3 mV dec^−1^, respectively, although they are slightly higher than the Tafel slope of 28.3 mV dec^−1^ in 1 M KOH DI water. The above analyses indicate that the utilization of seawater to prepare the alkaline electrolyte mildly affects the reaction kinetics of HW-NiMoN-2h and that the main cause of this effect is the introduction of Na^+^ ions. The HER performance of selected prepared catalyts was measured in 1 M KOH seawater and the results are shown in Fig. 6c. Additionally, the overpotentials required by these catalysts to drive current densities of 100, 500, and 1000 mA cm^−2^ are summarized in Table S5. Specifically, based on a comparison between Tables S3 and S5, the overpotential difference at 1000 mA cm^−2^ between 1 M KOH DI water and 1 M KOH seawater for HW-NiMoN-2h is 23 mV, smaller than the corresponding overpotential differences of 29 and 65 mV for HT-NiMoN and WB-NiMoN, respectively. Figure S20 shows the Tafel slopes for different catalysts in 1 M KOH seawater. Based on a comparison between Figs. 3e and S20, the Tafel slopes for HT-NiMoN, WB-NiMoN, and Pt/C clearly increased from fresh water to seawater while that for HW-NiMoN-2h remained nearly unchanged. Given the similar intrinsic activity among different NiMoN samples, the above results indicate that the catalyst with a larger specific surface area is less sensitive to the poisoning effect of impurities from seawater.

Although faradaic efficiency (FE) for HER in seawater is not a significant concern like it is for OER under the same condition, the FE of HW-NiMoN-2h paired with an OER catalyst was measured based on a thoroughly discussed drainage method to confirm that no side reaction would occur during the seawater H_2_ production [32, 61]. NiFe LDH prepared via a previously reported method was employed as the OER catalyst for seawater oxidation, and its OER performance is shown in Fig. S21a [61]. NiFe LDH was found to deliver a current density of 1000 mA cm^−2^ at an overpotential of 328 mV in 1 M KOH seawater, much lower than the thermodynamic potential of Cl^−^/ClO^−^, indicating that the chloride oxidation reaction can be thermodynamically suppressed in 1 M KOH seawater [62]. NiFe LDH was then paired with HW-NiMoN-2h for overall seawater electrolysis, and performance of the pair is shown in Fig. S21b. Finally, FE measurements were carried out at 500 mA cm^−2^ with an electrode area of 0.5 cm^2^ in the two-electrode setup shown in Fig. S22. As shown in Figs. 6d and S22, the experimentally determined amounts of H_2_ and of O_2_ produced are in good agreement with their theoretical values, which proves that the FE for both HER and OER in 1 M KOH seawater was close to 100% and that no side reaction occurred during the seawater H_2_ production.

Catalyst and electrode stability in seawater is more challenging than in fresh water. The Cl^−^ in seawater will continuously corrode the metal substrate and deactivate the electrode by destroying its structural integrity [63]. Kuang et al*.* [64] reported that Ni foam without a protective layer experienced severe corrosion in a 1 M KOH 2 M NaCl electrolyte within merely 8 h. However, the Zeta potentials shown in Fig. S23 indicate the negative charge of different NiMoN samples, with HW-NiMoN-2h exhibiting most negative Zeta potential of -16.0 mV. Such a strongly negative charge allows HW-NiMoN-2h to effectively repel Cl^−^ anions, reducing the corrosion from Cl^−^. The stability of HW-NiMoN-2h in seawater was evaluated via CP testing as shown in Fig. 6e. At 500 mA cm^−2^, the potential changed from − 91 to − 100 mV versus RHE over 100 h of CP testing, resulting in a degradation rate of 0.09 mV h^−1^. After CP testing at 500 mA cm^−2^, the XRD pattern for HW-NiMoN-2h was obtained, and the result in Fig. S24 shows that its crystal structure remained the same as before the CP test. To reveal the real active species after the stability test, XPS spectra for HW-NiMoN-2h were obtained both immediately after stability testing and after 9 min of Ar plasma sputtering to remove the surface contamination and the oxidized layer produced during the stability test. In Fig. S25, the XPS spectra before Ar plasma sputtering show oxidized metal states, similar to the corresponding results in Fig. S13, and the higher bonding energy of M–N in comparison to that shown in Fig. 2e. Following the subsequent Ar plasma sputtering, Ni^0^, Mo^3+^, M–N, and N–H can be observed in the XPS results, indicating that the real active species remained the same even after 100 h of CP testing at 500 mA cm^−2^ in 1 M KOH seawater. TEM analysis was also conducted on HW-NiMoN-2h after the stability test. The HRTEM and SAED images respectively displayed in Fig. S26a, b show lattice spaces and diffraction rings, respectively, corresponding to Ni and Ni_0.2_Mo_0.8_N, and the EDS mapping shown in Fig. S26c–f indicates the uniform distribution of all the elements after the stability test. During CP testing of HW-NiMoN-2h at 1000 mA cm^−2^, the overpotential exhibited a small increase of 8 mV over 70 h, corresponding to a degradation rate of about 0.114 mV h^−1^, which is slightly higher than that of 0.09 mV h^−1^ at 500 mA cm^−2^ due to the higher rate of H_2_ production. The HER performance of HW-NiMoN-2h was also systematically compared to that of previously reported hierachical HER catalysts in seawater-based electrolytes and the details are provided in Table S6, demonstrating the state-of-the-art HER activity and stability of HW-NiMoN-2h for seawater electrolysis.

## Conclusion

Here we successfully synthesized hierarchical interconnected NiMoN nanorod-nanowire clusters on the surface of NF based on a rational combination of hydrothermal and water bath methods. The similarity in crystal structure among NiMoO_4_ samples prepared using different methods is key to the formation of the hierarchical interconnected morphology. ECSA and BET data showed that the hierarchical HW-NiMoN-2h has a larger specific area than either HT-NiMoN or WB-NiMoN prepared using the hydrothermal or water bath method, respectively. With intrinsic activity similar to that of these other NiMoN samples but more active sites, HW-NiMoN-2h required an overpotential of only 107 mV to deliver a current density of 1000 mA cm^−2^ in 1 M KOH DI water, outperforming HT-NiMoN, WB-NiMoN, and the benchmark Pt/C. Additionally, its interconnected characteristics endowed HW-NiMoN-2h with higher mechanical strength, allowing it to withstand 30 min sonication, which almost completely destroyed the structural integrity of both the HT-NiMoN and WB-NiMoN samples. Due to its increased mechanical strength, HW-NiMoN-2h exhibited excellent stability at 500 mA cm^−2^ in 1 M KOH DI water and significantly outperformed both HT-NiMoN and WB-NiMoN under the rigorous current density of 1000 mA cm^−2^ in 1 M KOH DI water. A comparison of the HER performance of HW-NiMoN-2h in alkaline fresh water and simulated and natural seawater suggested that the degradation of its catalytic activity in alkaline seawater mainly resulted from the addition of Na^+^. However, in 1 M KOH seawater, HW-NiMoN-2h mostly retained its outstanding HER performance in alkaline fresh water, with LSV results showing that it achieved a current density of 1000 mA cm^−2^ at a potential of − 130 mV versus RHE. With its larger specific surface area, HW-NiMoN-2h exhibited less sensitivity to Na^+^ than other NiMoN samples. CP test results showed that the degradation rates of HW-NiMoN-2h at 500 and 1000 mA cm^−2^ were 0.09 and 0.114 mV h^−1^, respectively, illustrating its excellent stability for seawater H_2_ production. This study proposed a general strategy of building hierarchical interconnected structures to increase the active sites in nanocatalysts while at the same time strengthening their mechanical stability, which we hope will inspire the academic and industrial communities to design and apply highly active and robust nanocatalysts.

### Supplementary Information

Below is the link to the electronic supplementary material.

Supplementary file1 (PDF 2935 KB)
