# Supplementary material for: Hierarchical Interconnected NiMoN with Large Specific Surface Area and High Mechanical Strength for Efficient and Stable Alkaline Water/Seawater Hydrogen Evolution
Source: Nanomicro Lett. 2023 Jun 19;15:157. doi: 10.1007/s40820-023-01129-y (PMC10279610; doi:10.1007/s40820-023-01129-y)
Supplement: Supplementary file 1 — Supplementary file1 (PDF 2935 KB) [file 40820_2023_1129_MOESM1_ESM.pdf]

Supporting Information for

Hierarchical Interconnected NiMoN with Large Specific Surface Area and High Mechanical Strength for Efficient and Stable Alkaline Water/Seawater Hydrogen Evolution

Minghui Ning[1], Yu Wang[2], Libo Wu[2], Lun Yang[3], Zhaoyang Chen[4], Shaowei Song[1], Yan Yao[4], Jiming Bao[4], Shuo Chen[1,] * and Zhifeng Ren[1,] *

[1]Department of Physics and Texas Center for Superconductivity at the University of Houston (TcSUH), University of Houston, Houston, TX 77204, USA

[2]Cullen College of Engineering and TcSUH, University of Houston, Houston, TX 77204, USA

[3]School of Materials Science and Engineering, Hubei Normal University, Huangshi, Hubei 435002, P. R. China

[4]Department of Electrical and Computer Engineering and TcSUH, University of Houston, Houston, TX 77204, USA

*Corresponding authors. E-mail: schen34@uh.edu (Shuo Chen), zren@uh.edu (Zhifeng Ren)

Supplementary Figures and Tables

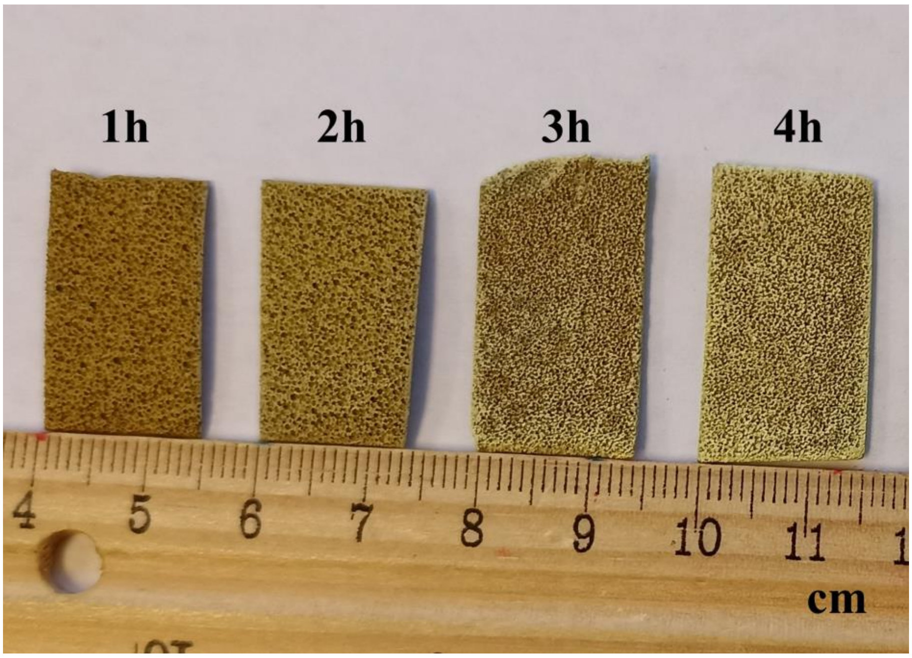

Fig. S1 Image of HW-$NiMoO_4$ prepared with different amounts of water bath reaction time

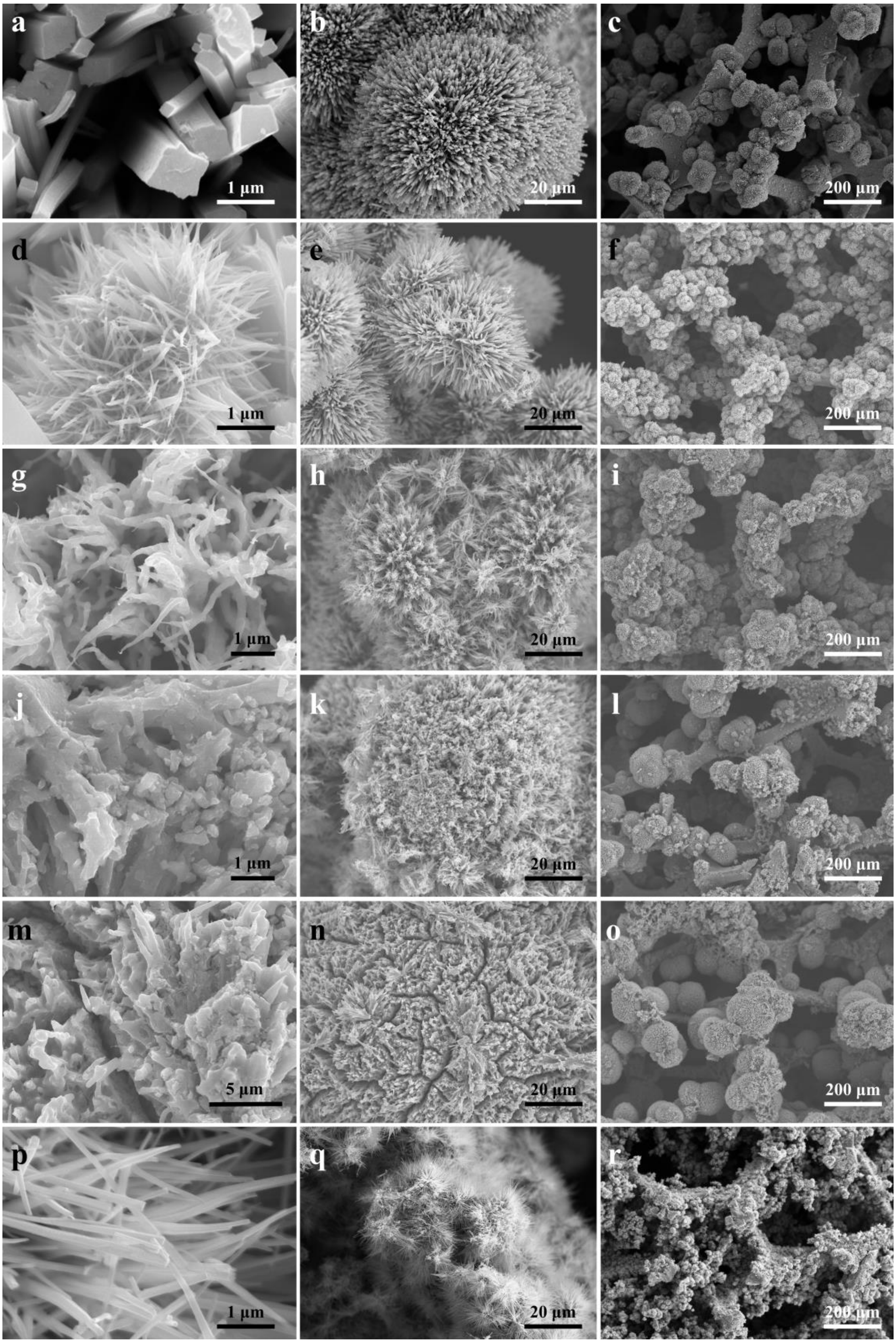

Fig. S2 SEM images at different resolutions for **a-c** HT-NiMoN, **d-f** HW-NiMoN-1h, **g-i** HW-NiMoN-2h, **j-l** HW-NiMoN-3h, **m-o** HW-NiMoN-4h, and **p-r** WB-NiMoN

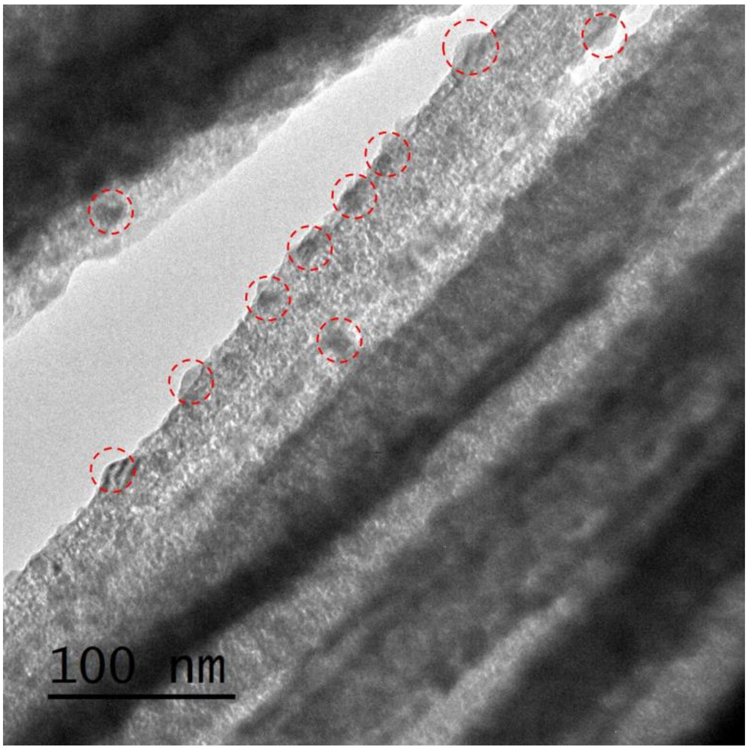

Fig. S3 TEM image of HW-NiMoN-2h. Dashed circles indicate the presence of nanodots on the surfaces of the NiMoN nanowires

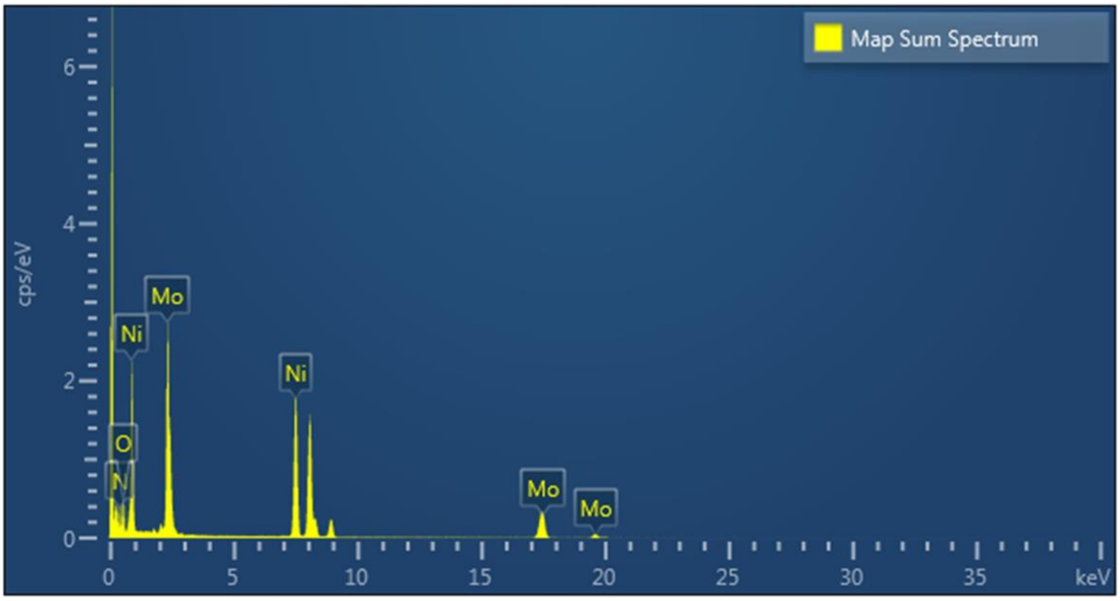

Element	Wt%	Wt% Sigma	Atomic %
Ni	34.71	0.42	28.47
Mo	51.22	0.54	25.71
N	8.12	0.40	27.91
O	5.95	0.24	17.91

Fig. S4 EDS point analysis for HW-NiMoN-2h

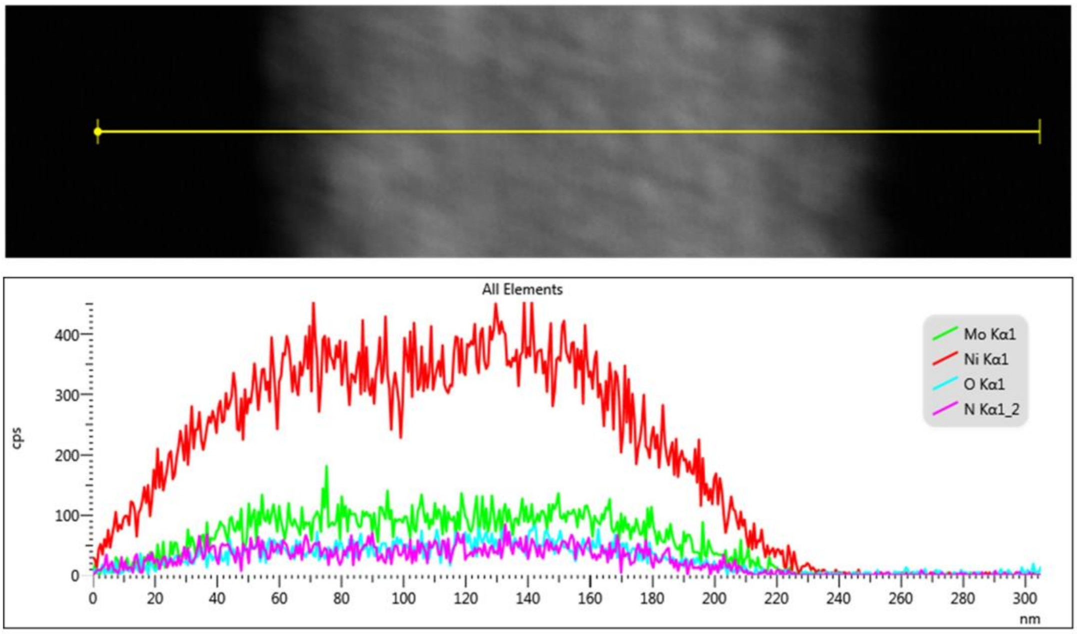

Fig. S5 EDS linear scan of HW-NiMoN-2h

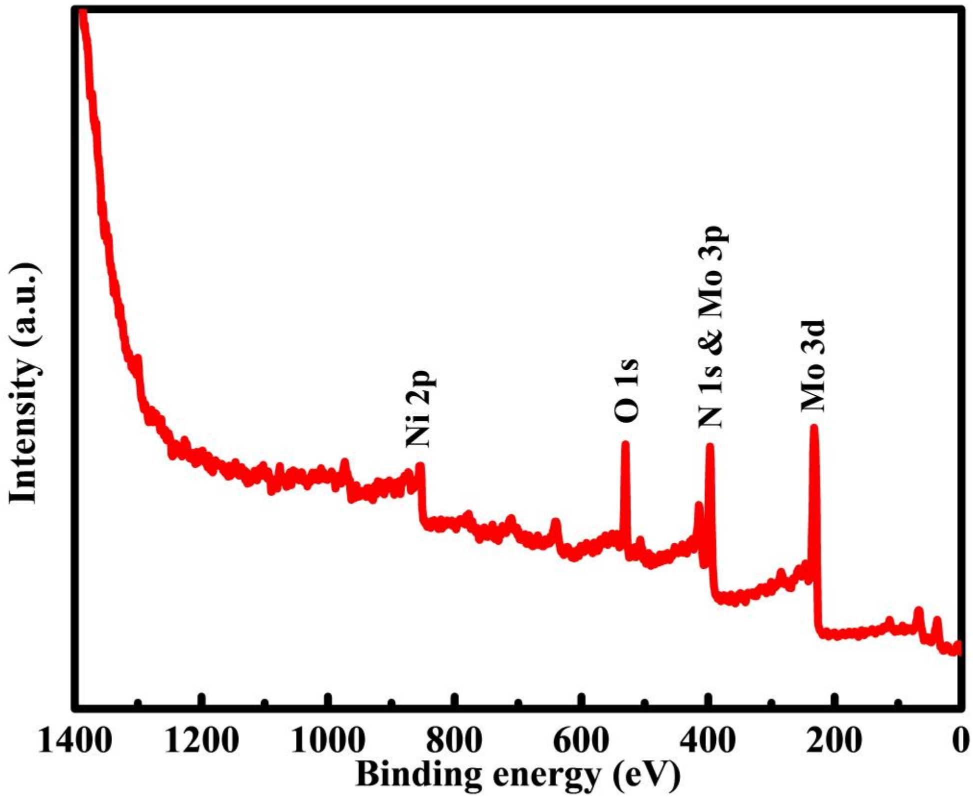

Fig. S6 XPS survey spectrum for HW-NiMoN-2h

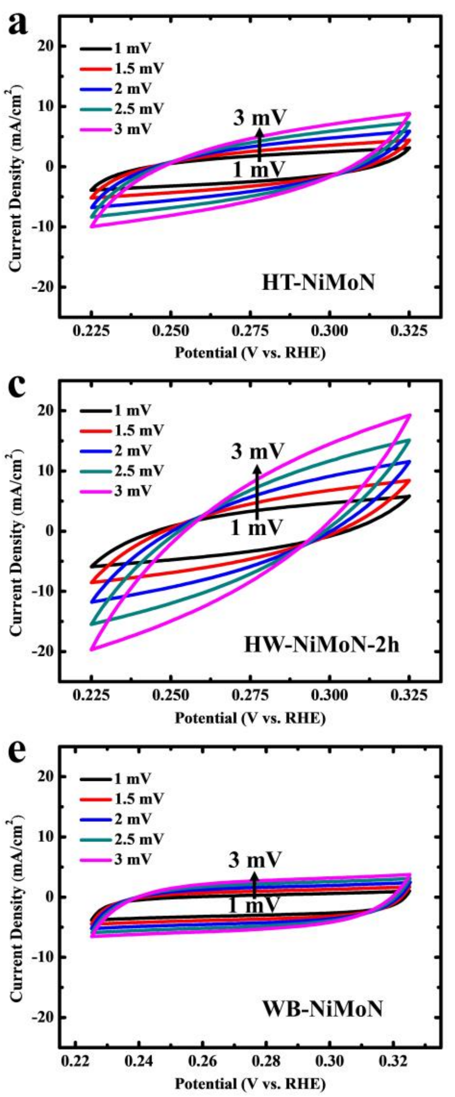

Fig. S7 CV measurements at different scan rates for **a** HT-NiMoN, **b** HW-NiMoN-1h, **c** HW-NiMoN-2h, **d** HW-NiMoN-3h, and **e** WB-NiMoN

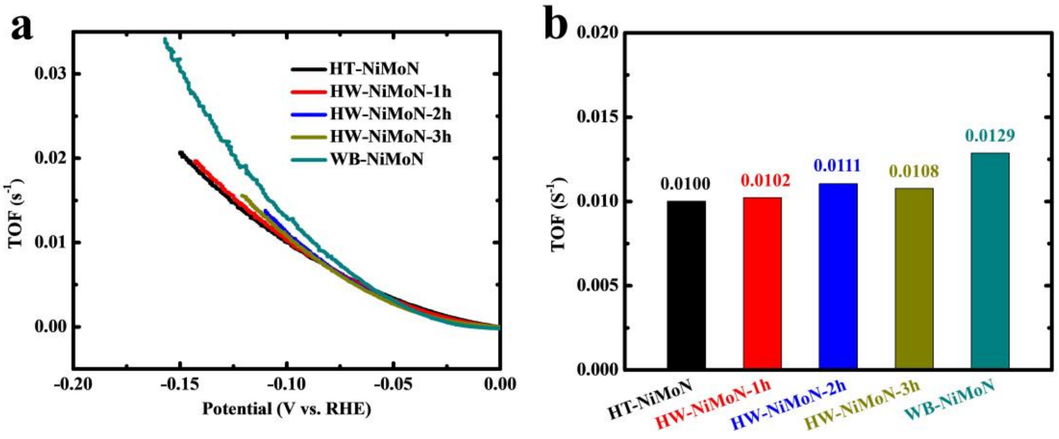

Fig. S8 TOF **a** plots and **b** values at -0.1 V *vs.* RHE for selected NiMoN samples

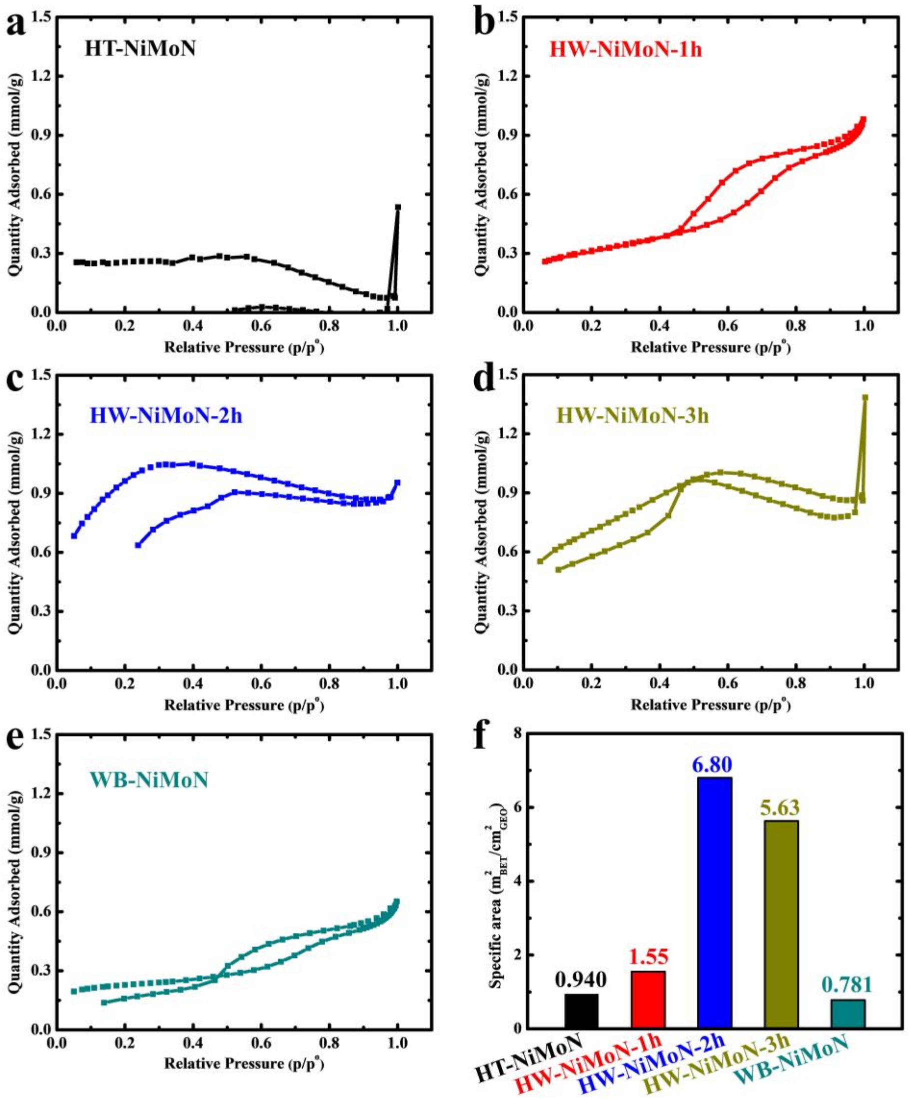

Fig. S9 BET measurements of **a** HT-NiMoN, **b** HW-NiMoN-1h, **c** HW-NiMoN-2h, **d** HW-NiMoN-3h, and **e** WB-NiMoN. **f** BET specific area values for NiMoN samples prepared using different methods

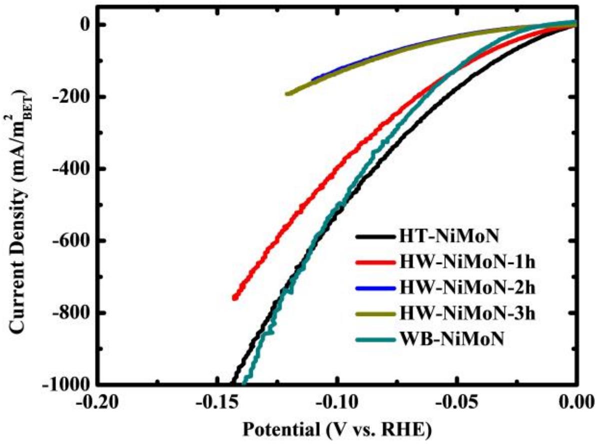

Fig. S10 HER activity of NiMoNs normalized by the BET specific area

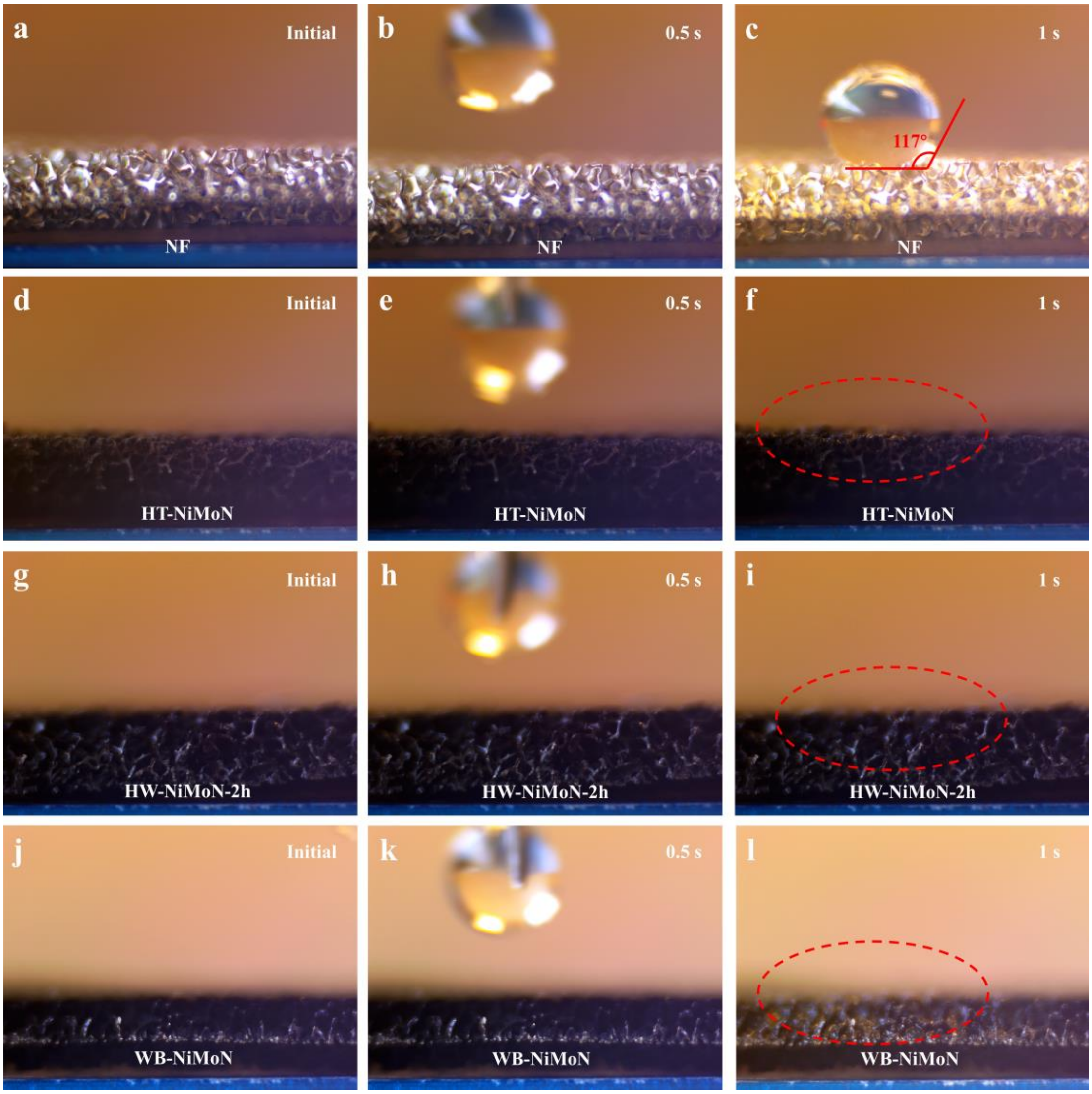

Fig. S11 Contact angle tests of DI water dripped onto various catalysts. **a-c** NF, **d-f** HT-NiMoN, **g-i** HW-NiMoN-2h, and **j-l** WB-NiMoN

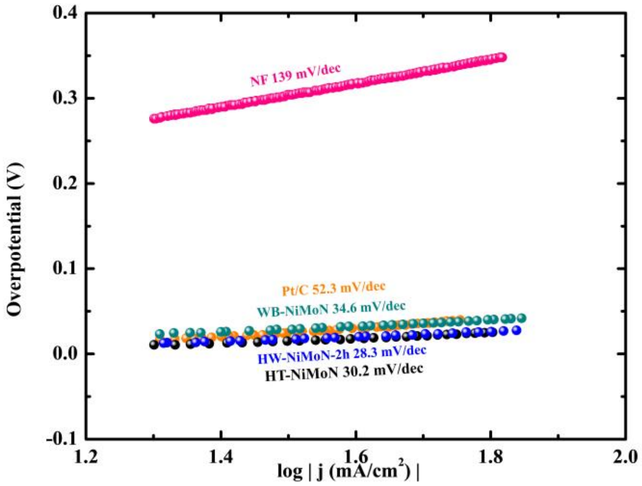

Fig. S12 Tafel slopes for different catalysts, including NF

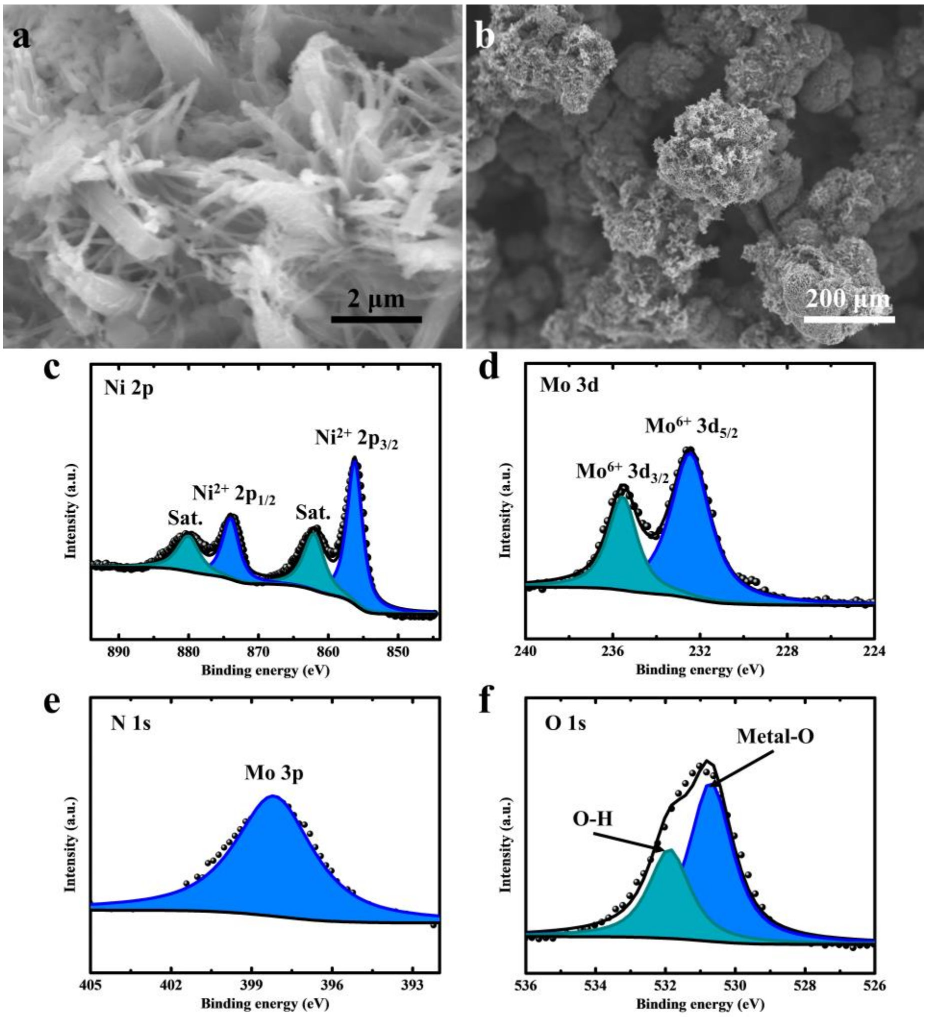

Fig. S13 a-b SEM images of HW-NiMoN-2h after CP testing at 500 mA/cm^2 in 1 M KOH DI water for 100 h. XPS spectra of **c** Ni 2p, **d** Mo 3d, **e** N 1s, and **f** O 1s for HW-NiMoN-2h after CP testing at 500 mA/cm^2 in 1 M KOH DI water for 100 h

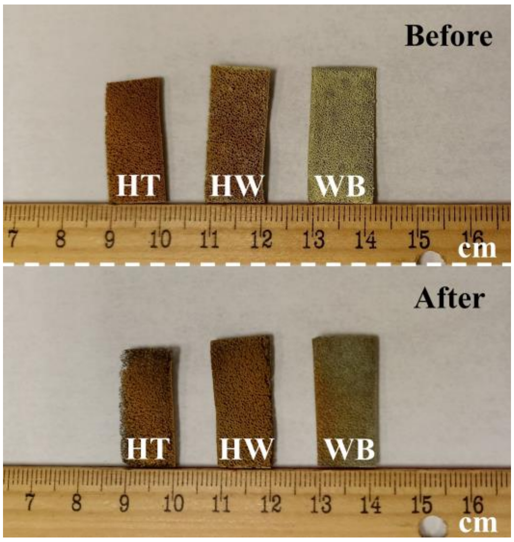

Fig. S14 Images of HT-$NiMoO_4$, HW-$NiMoO_4$-2h, and WB-$NiMoO_4$ before and after 30 min sonication

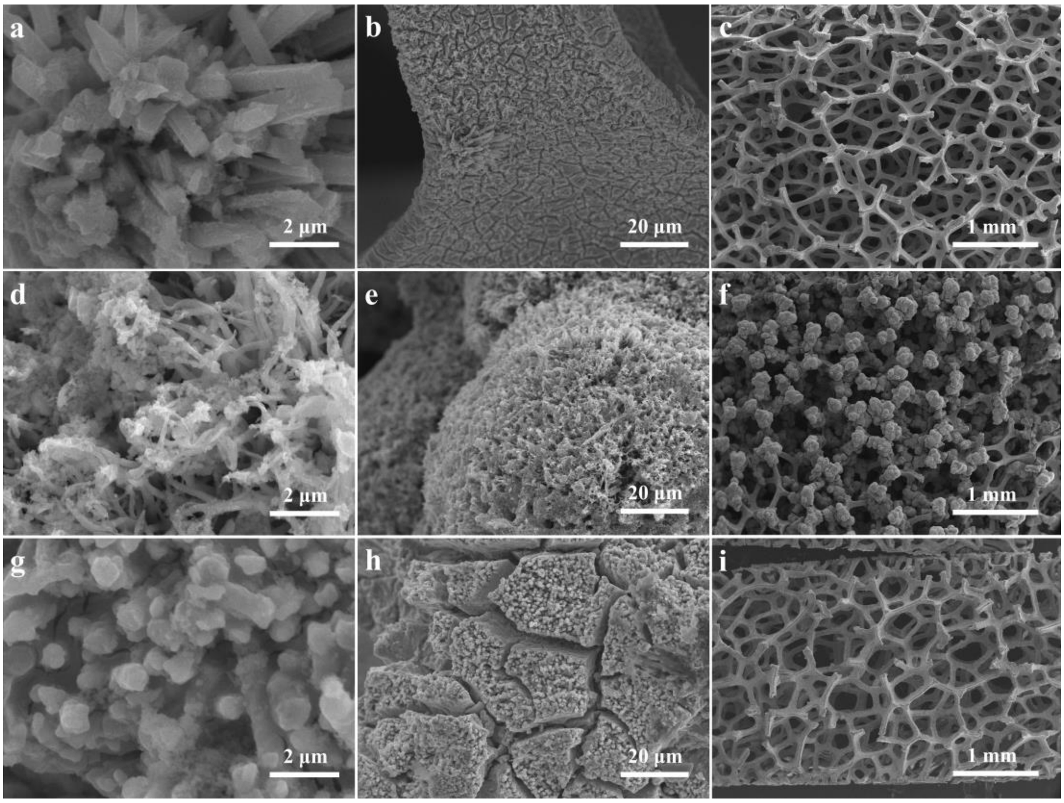

Fig. S15 SEM images of **a-c** HT-NiMoN, **d-f** HW-NiMoN-2h, and **g-i** WB-NiMoN after 30 min sonication

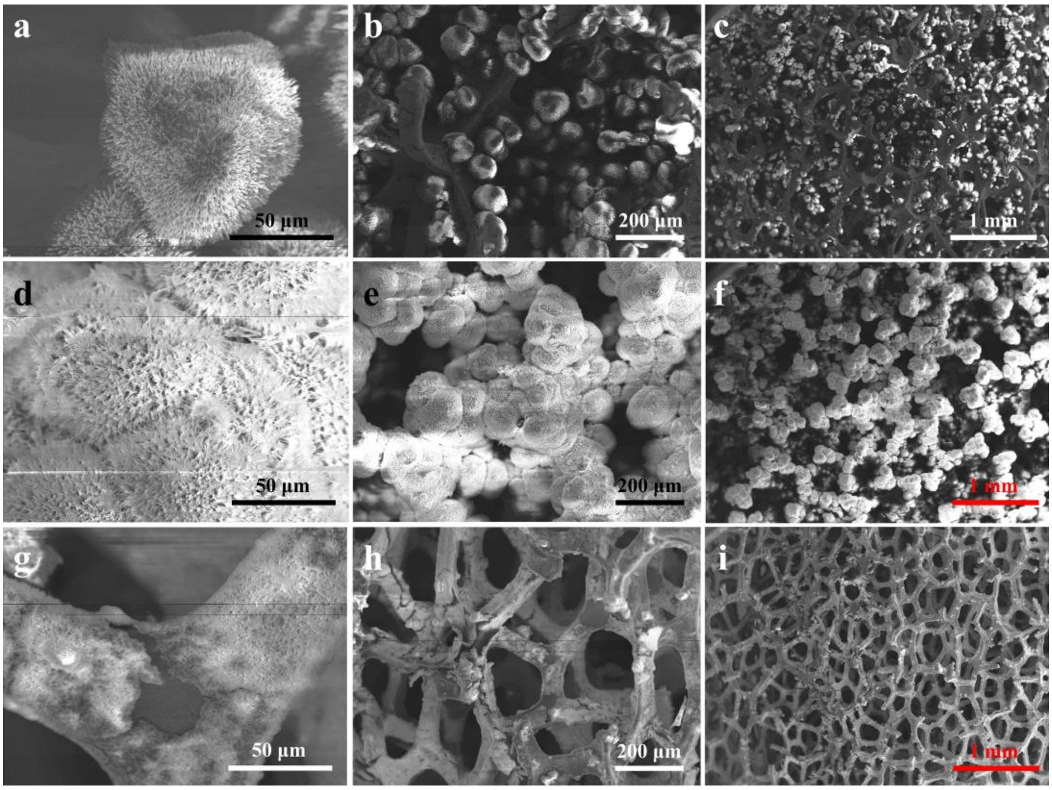

Fig. S16 SEM images of **a-c** HT-$NiMoO_4$, **d-f** HW-$NiMoO_4$-2h, and **g-i** WB-$NiMoO_4$ after 30 min sonication

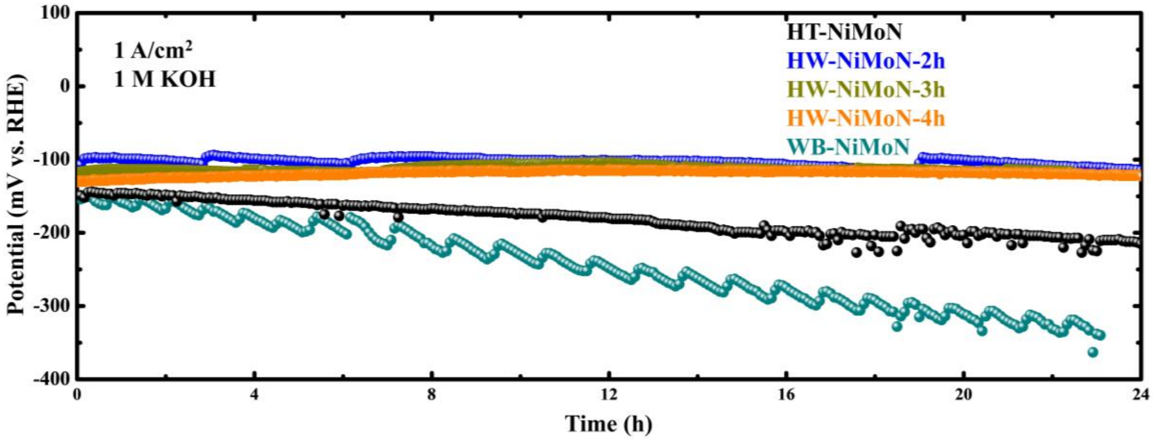

Fig. S17 Chronopotentiometric tests of HT-NiMoN, HW-NiMoN-2h, HW-NiMoN-3h, HW-NiMoN-4h, and WB-NiMoN at 1 A/cm^2 in 1 M KOH DI water

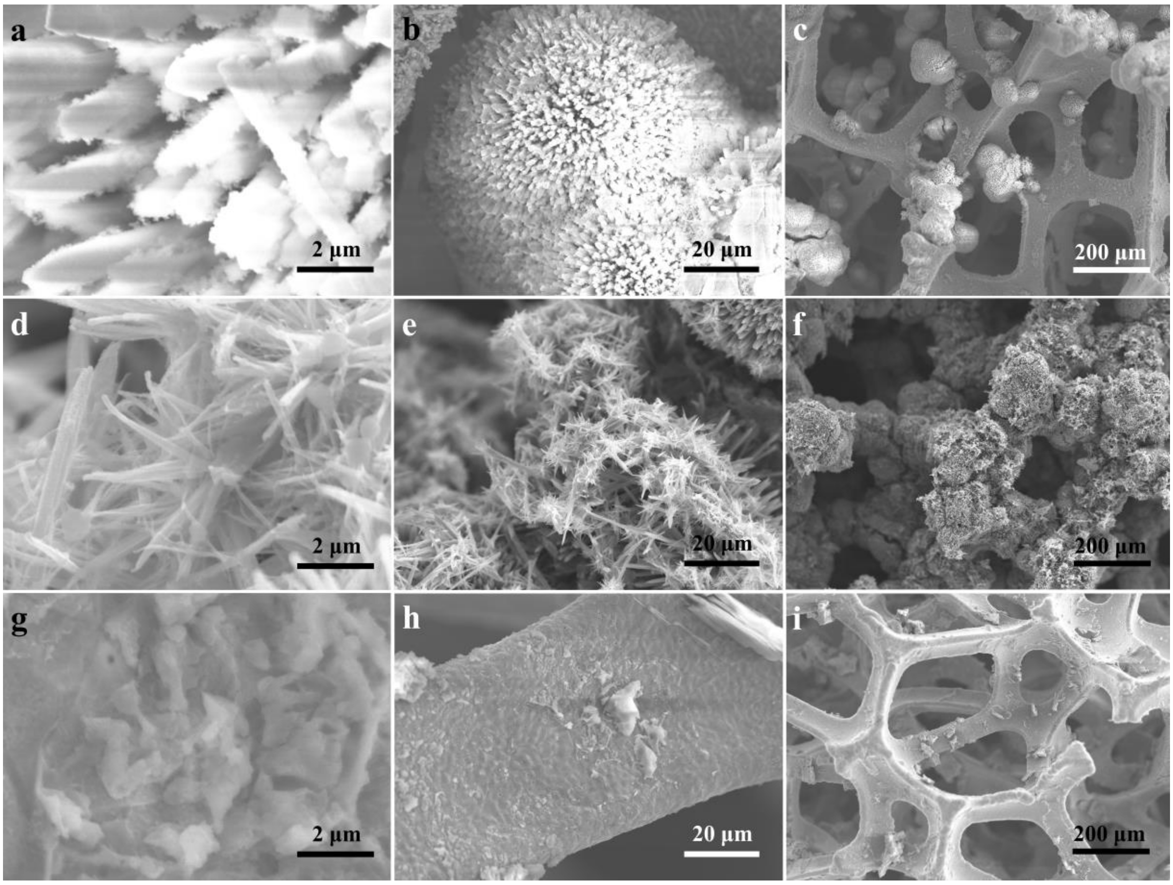

Fig. S18 SEM images of **a-c** HT-NiMoN, **d-f** HW-NiMoN-2h, and **g-i** WB-NiMoN after CP testing at 1 A/cm^2 in 1 M KOH DI water for 24 h, 24 h, and 23 h, respectively

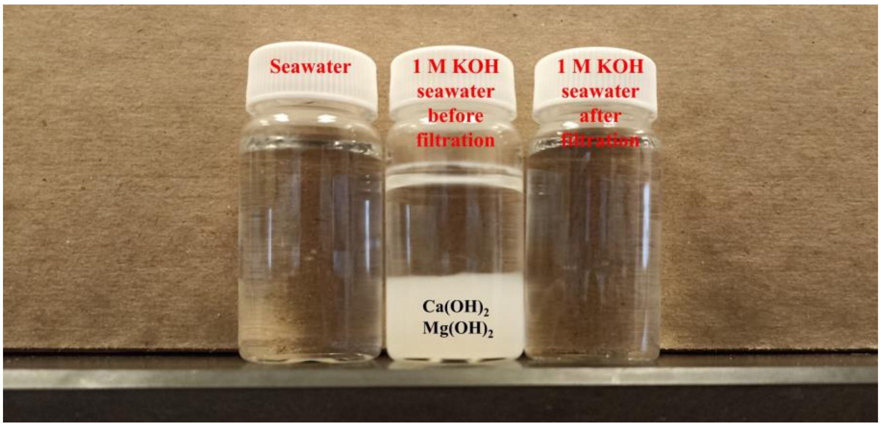

Fig. S19 Image of seawater and of 1 M KOH seawater before and after filtration

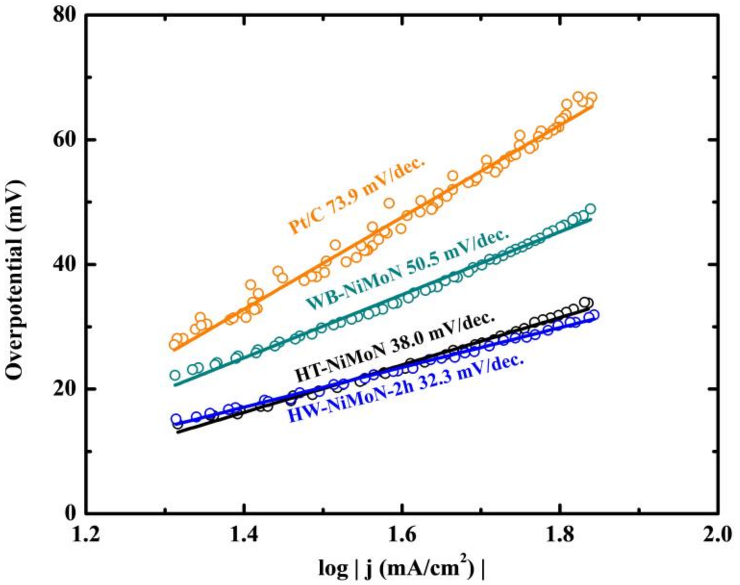

Fig. S20 Tafel slopes for HT-NiMoN, HW-NiMoN-2h, WB-NiMoN, and Pt/C in 1 M KOH seawater

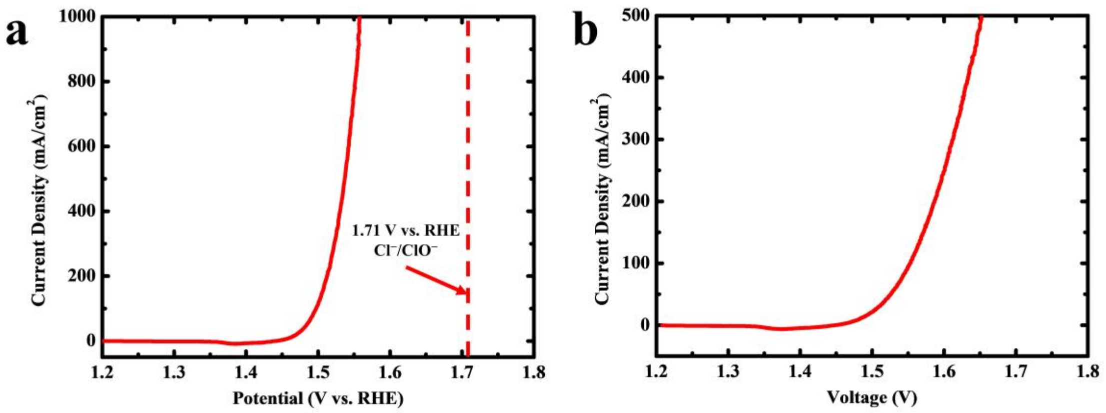

Fig. S21 **a** OER performance of NiFe LDH and **b** overall performance of NiFe LDH||HW-NiMoN-2h in 1 M KOH seawater

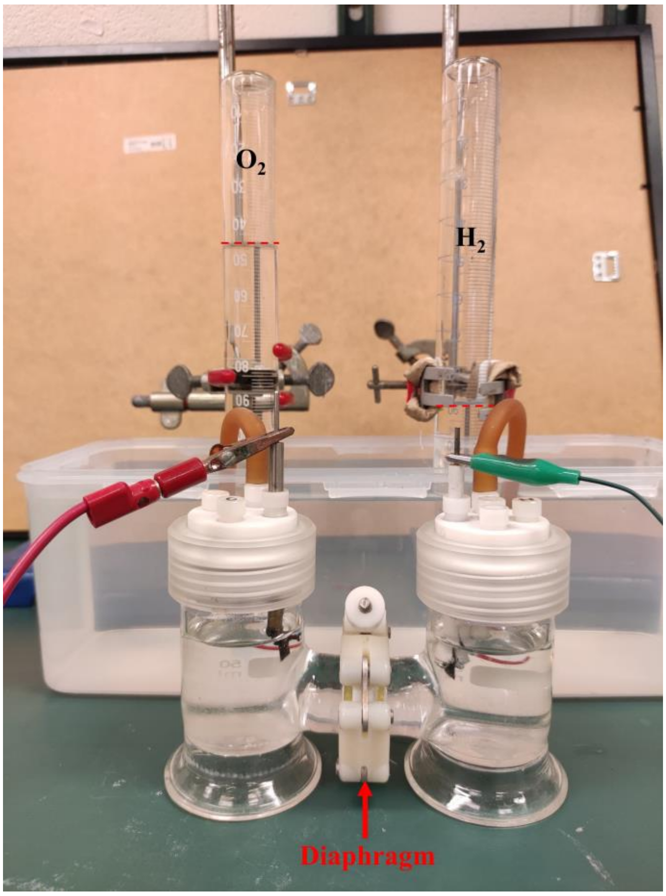

Fig. S22 Drainage setup for FE measurements based on the H-type electrolyzer. Dashed lines indicate H_2 and O_2 production after 50 h

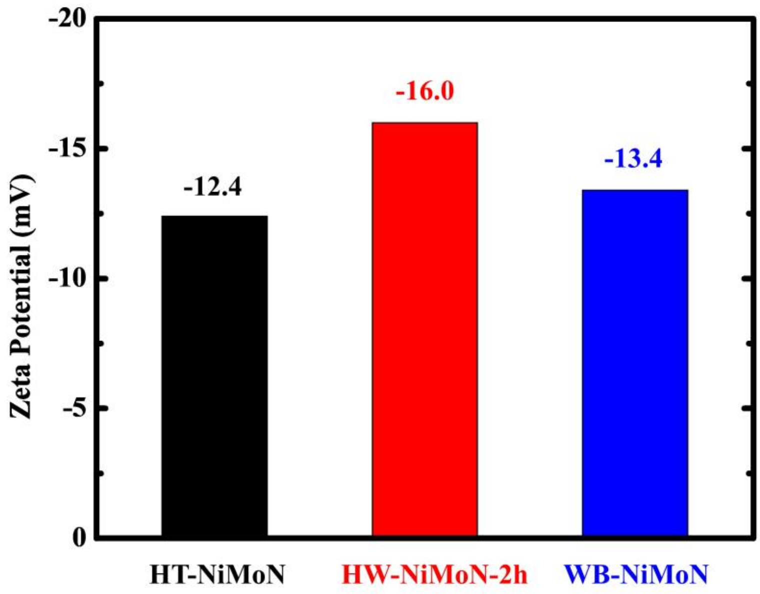

Fig. S23 Zeta potentials of different NiMoN samples in DI water

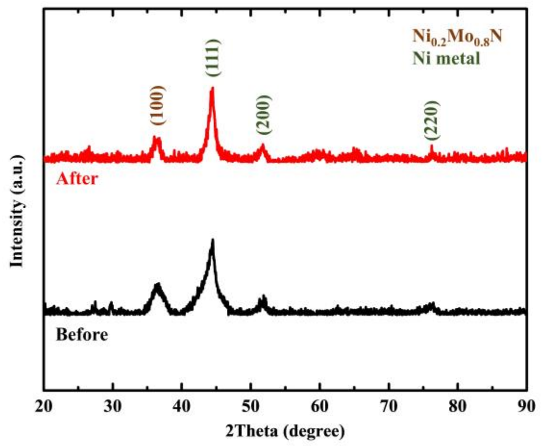

Fig. S24 XRD measurements before and after 100 h of CP testing at 500 mA/cm^2 in 1 M KOH seawater

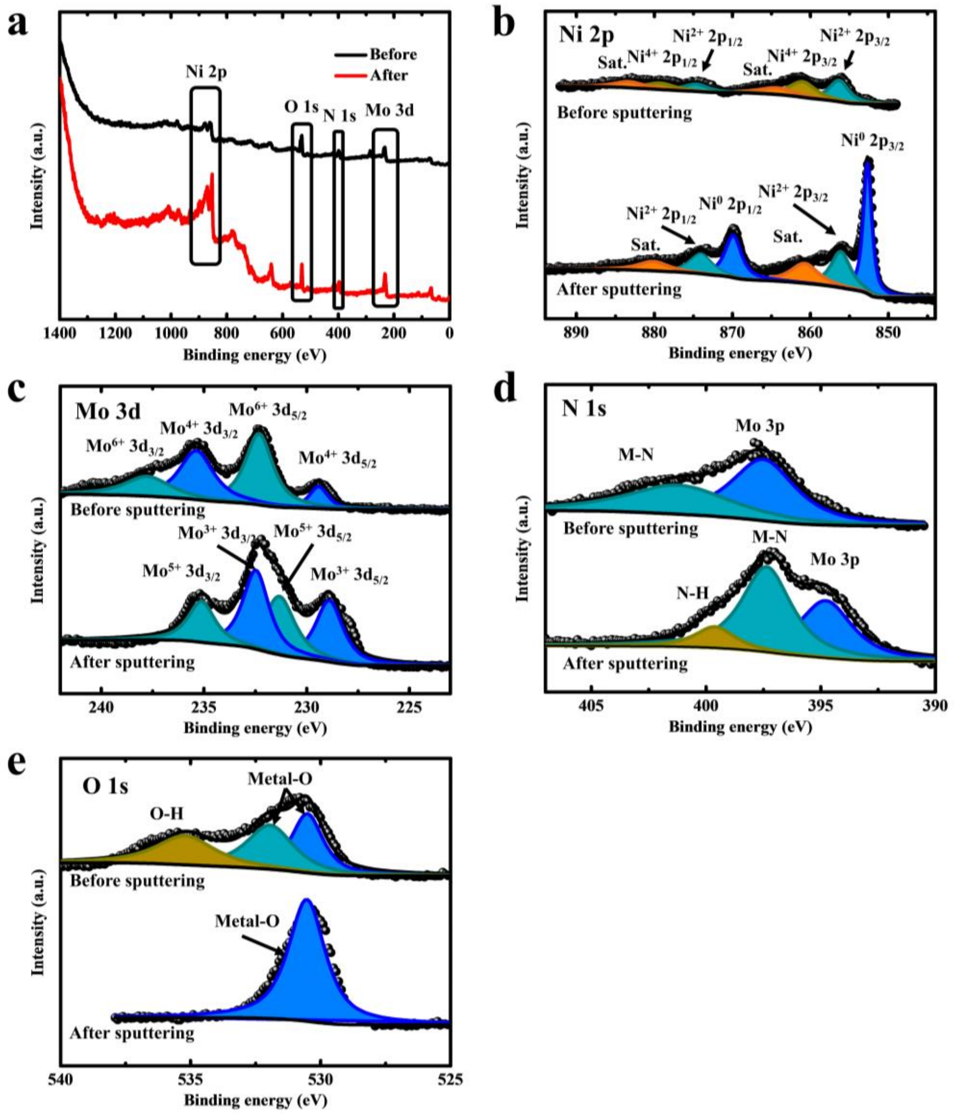

Fig. S25 XPS measurements before and after plasma sputtering of HW-NiMoN-2h subsequent to 100 h of CP testing at 500 mA/cm^2 in 1 M KOH seawater. **a** Survey spectra and spectra of **b** Ni 2p, **c** Mo 3d, **d** N 1s, and **e** O 1s

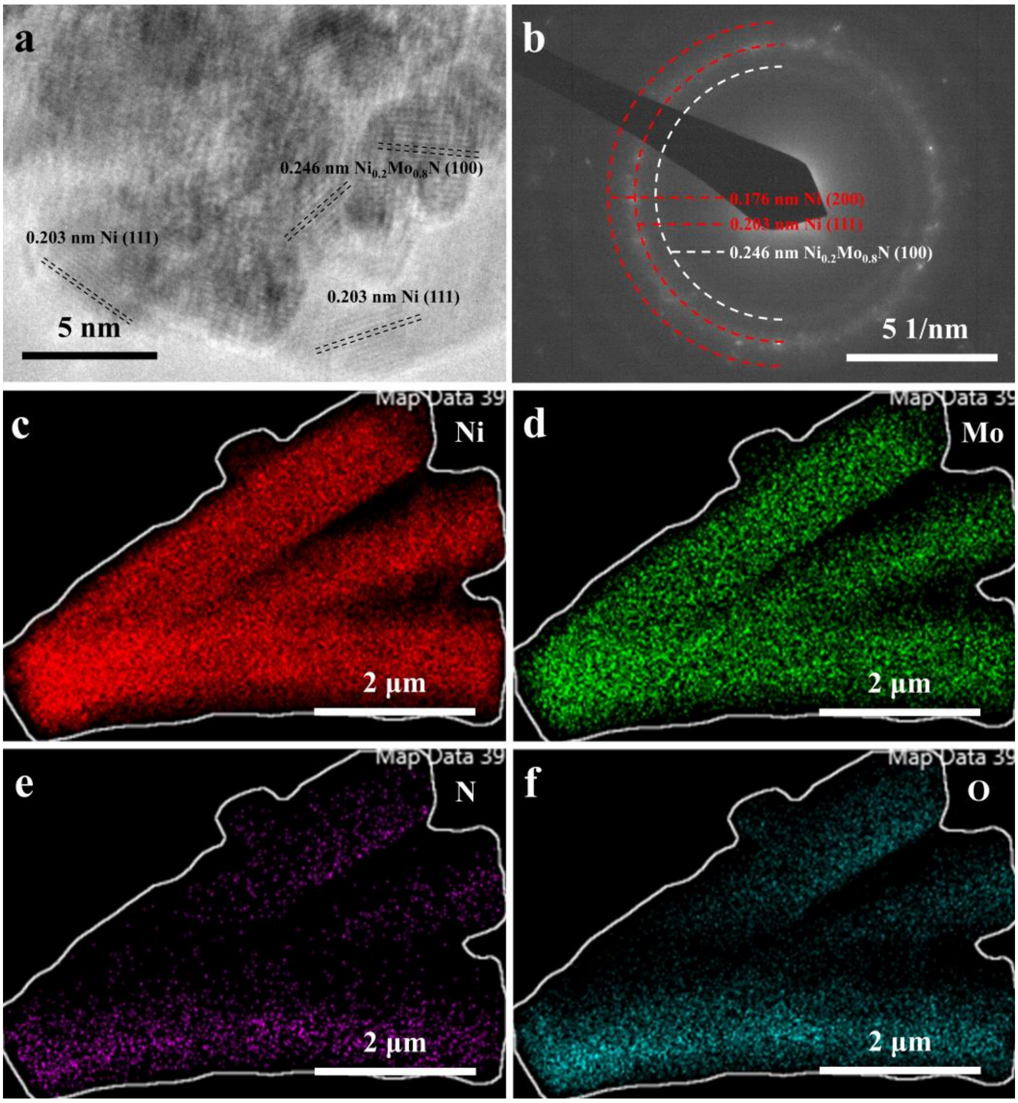

Fig. S26 a HRTEM and **b** SAED images and EDS mapping of **c** Ni, **d** Mo, **e** N, and **f** O, for HW-NiMoN-2h after 100 h of CP testing at 500 mA/cm^2 in 1 M KOH seawater

Table S1 ICP-OES results for HW-NiMoN-2h

HW-NiMoN-2h	Wt%
Ni	28.35
Mo	41.65

Table S2 BET area, mass density, and specific area values for NiMoN samples prepared using different methods

Sample	HT-NiMoN	HW-NiMoN-1h	HW-NiMoN-2h	HW-NiMoN-3h	WB-NiMoN
BET area (m^2/g)	17.5	23.9	74.6	54.8	16.4
Mass density (mg/cm$^2_{GEO}$)	53.7	64.8	91.1	103	47.6
Specific area (m$^2_{BET}$/cm$^2_{GEO}$)	0.940	1.55	6.80	5.63	0.781

Table S3 Overpotentials required by different catalysts to drive various current densities in 1 M KOH DI water

Overpotential (mV)	HT-NiMoN	HW-NiMoN-2h	WB-NiMoN	Pt/C	NF
100 mA/cm^2	36	34	50	60	353
500 mA/cm^2	101	76	111	165	--
1000 mA/cm^2	149	107	155	230	--

Table S4 Overpotentials required by HW-NiMoN-2h to drive various current densities in different electrolytes

Overpotential (mV)	1 M KOH DI water	1 M KOH 0.5 M NaCl	1 M KOH seawater
100 mA/cm^2	34	38	40
500 mA/cm^2	76	88	91
1000 mA/cm^2	107	127	130

Table S5 Overpotentials required by different catalysts to drive various current densities in 1 M KOH seawater

Overpotential (mV)	HT-NiMoN	HW-NiMoN-2h	WB-NiMoN	Pt/C	NF
100 mA/cm^2	44	40	62	85	376
500 mA/cm^2	124	91	161	199	--
1000 mA/cm^2	178	130	220	259	--

Table S6 HER performance of different hierarchical catalysts in seawater electrolyte

Catalyst	Electrolyte	Activity	Stability	Refs.
HW-NiMoN-2h	1 M KOH seawater	1 A/cm^2 at -0.13 V *vs.* RHE	70 h at 1 A/cm^2	This work
Ni-MoN	1 M KOH seawater	1 A/cm^2 at -0.176 V *vs.* RHE	200 h at 500 mA/cm^2	[S1]
PF-NiCoP/NF	Natural seawater	10 mA/cm^2 at -0.287 V *vs.* RHE	20 h at 10 mA/cm^2	[S2]
NiCoN|Ni_xP|NiCoN	Natural seawater	10 mA/cm^2 at -0.165 V *vs.* RHE	24 h at 10 mA/cm^2	[S3]
Ni_2P-CoOOH	Simulated seawater	100 mA/cm^2 at -0.4 V *vs.* RHE	100 h at ~100 mA/cm^2	[S4]
Co-Fe_2P	1 M KOH 0.5 M NaCl	100 mA/cm^2 at -0.221 V *vs.* RHE	22 h at 250 mA/cm^2	[S5]
1D-Cu@Co-CoO/Rh	1 M KOH 0.5 M NaCl	50 mA/cm^2 at -0.2424 V *vs.* RHE	12 h at 10 mA/cm^2	[S6]
$MoNi_4/MoO_{3-x}$/NiCo	Simulated seawater	10 mA/cm^2 at -0.101 V *vs.* RHE	20 h at ~21 mA/cm^2	[S7]

NiCoFeP@NiCoP	Simulated seawater	10 mA/cm^2 at -0.4 V *vs.* RHE	10 h at ~25 mA/cm^2	[S8]
CoNiP/Co_xP	Natural seawater	10 mA/cm^2 at -0.29 V *vs.* RHE	500 h at 10 mA/cm^2	[S9]
Co@RuCo-3	1 M KOH seawater	10 mA/cm^2 at -0.059 V *vs.* RHE	100 h at 50 mA/cm^2	[S10]

Supplemenary References

[S1] L. Wu, F. Zhang, S. Song, M. Ning, Q. Zhu et al., Efficient alkaline water/seawater hydrogen evolution by a nanorod-nanoparticle-structured ni-mon catalyst with fast water-dissociation kinetics. Adv. Mater. **34**(21), 2201774 (2022). https://doi.org/10.1002/adma.202201774

[S2] Q. Lv, J. Han, X. Tan, W. Wang, L. Cao et al., Featherlike nicop holey nanoarrys for efficient and stable seawater splitting. ACS Appl. Energy Mater. **2**(5), 3910-3917 (2019). https://doi.org/10.1021/acsaem.9b00599

[S3] L. Yu, L. Wu, S. Song, B. McElhenny, F. Zhang et al., Hydrogen generation from seawater electrolysis over a sandwich-like nicon|nixp|nicon microsheet array catalyst. ACS Energy Lett. **5**(8), 2681-2689 (2020). https://doi.org/10.1021/acsenergylett.0c01244

[S4] S. Zhang, W. Wang, F. Hu, Y. Mi, S. Wang et al., 2D coooh sheet-encapsulated ni(2)p into tubular arrays realizing 1000 ma cm(-2)-level-current-density hydrogen evolution over 100 h in neutral water. Nano-Micro Lett. **12**(1), 140 (2020). https://doi.org/10.1007/s40820-020-00476-4

[S5] S. Wang, P. Yang, X. Sun, H. Xing, J. Hu et al., Synthesis of 3d heterostructure co-doped fe2p electrocatalyst for overall seawater electrolysis. Appl. Catal. B-Environ. **297**, 120386 (2021). https://doi.org/10.1016/j.apcatb.2021.120386

[S6] P.K.L. Tran, D.T. Tran, D. Malhotra, S. Prabhakaran, D.H. Kim et al., Highly effective freshwater and seawater electrolysis enabled by atomic rh-modulated co-coo lateral heterostructures. Small **17**(50), 2103826 (2021). https://doi.org/10.1002/smll.202103826

[S7] Y. Zhang, G. Yan, Y. Shi, H. Tan, Y. Li, A branch-leaf-like hierarchical self-supporting electrode as a highly efficient catalyst for hydrogen evolution. New J. Chem. **45**(24), 10890-10896 (2021). https://doi.org/10.1039/d1nj00836f

[S8] G. Yan, H. Tan, Y. Wang, Y. Li, Amorphous quaternary alloy phosphide hierarchical nanoarrays with pagoda-like structure grown on ni foam as ph-universal electrocatalyst for hydrogen evolution reaction. Appl. Surf. Sci. **489**, 519-527 (2019). https://doi.org/10.1016/j.apsusc.2019.05.254

[S9] D. Liu, H. Ai, M. Chen, P. Zhou, B. Li et al., Multi-phase heterostructure of conip/coxp for enhanced hydrogen evolution under alkaline and seawater

conditions by promoting h2o dissociation. Small **17**(17), 2007557 (2021). https://doi.org/10.1002/smll.202007557

[S10] H. Huang, H. Jung, C.-Y. Park, S. Kim, A. Lee et al., Surface conversion derived core-shell nanostructures of co particles@ruco alloy for superior hydrogen evolution in alkali and seawater. Appl. Catal. B-Environ. **315**, 121554 (2022). https://doi.org/10.1016/j.apcatb.2022.121554
